# Phosphorylated STAT5 directly facilitates parvovirus B19 DNA replication in human erythroid progenitors through interaction with the MCM complex

**DOI:** 10.1371/journal.ppat.1006370

**Published:** 2017-05-01

**Authors:** Safder S. Ganaie, Wei Zou, Peng Xu, Xuefeng Deng, Steve Kleiboeker, Jianming Qiu

**Affiliations:** 1 Department of Microbiology, Molecular Genetics and Immunology, University of Kansas Medical Center, Kansas City, Kansas, United States of America; 2 Department of Research and Development, Viracor Eurofins Laboratories, Lee’s Summit, Missouri, United States of America; Stony Brook University, UNITED STATES

## Abstract

Productive infection of human parvovirus B19 (B19V) exhibits high tropism for burst forming unit erythroid (BFU-E) and colony forming unit erythroid (CFU-E) progenitor cells in human bone marrow and fetal liver. This exclusive restriction of the virus replication to human erythroid progenitor cells is partly due to the intracellular factors that are essential for viral DNA replication, including erythropoietin signaling. Efficient B19V replication also requires hypoxic conditions, which upregulate the signal transducer and activator of transcription 5 (STAT5) pathway, and phosphorylated STAT5 is essential for virus replication. In this study, our results revealed direct involvement of STAT5 in B19V DNA replication. Consensus STAT5-binding elements were identified adjacent to the NS1-binding element within the minimal origins of viral DNA replication in the B19V genome. Phosphorylated STAT5 specifically interacted with viral DNA replication origins both *in vivo* and *in vitro*, and was actively recruited within the viral DNA replication centers. Notably, STAT5 interacted with minichromosome maintenance (MCM) complex, suggesting that STAT5 directly facilitates viral DNA replication by recruiting the helicase complex of the cellular DNA replication machinery to viral DNA replication centers. The FDA-approved drug pimozide dephosphorylates STAT5, and it inhibited B19V replication in *ex vivo* expanded human erythroid progenitors. Our results demonstrated that pimozide could be a promising antiviral drug for treatment of B19V-related diseases.

## Author summary

Human parvovirus B19 (B19V) infection can cause severe hematological disorders, a direct consequence of the death of infected human erythroid progenitor cells (EPCs) of the bone marrow and fetal liver. B19V replicates autonomously in human EPCs, and the erythropoietin (EPO) and EPO-receptor (EPO-R) signaling is required for productive B19V replication. The Janus kinase 2 (JAK2)-signal transducer and activator of transcription 5 (STAT5) signaling plays a key role in B19V replication. Here, we identify that phosphorylated STAT5 directly interacts with B19V replication origins and with minichromosome maintenance (MCM) complex in human EPCs, and that it functions as a scaffold protein to bring MCM to the viral replication origins and thus plays a key role in B19V DNA replication. Importantly, pimozide, a STAT5 phosphorylation-specific inhibitor and an FDA-approved drug, abolishes B19V replication in *ex vivo* expanded human EPCs; therefore, pimozide has the potential to be used as an antiviral drug for treatment of B19V-caused hematological disorders.

## Introduction

Human parvovirus B19 (B19V) is a small, non-enveloped parvovirus with a single-stranded (ss) DNA genome of 5.6 kb. It belongs to the genus *Erythroparvovirus* of the *Parvoviridae* family [1]. The B19V genome is flanked by identical inverted terminal repeats (ITRs) at both ends [2]. B19V is pathogenic to humans and causes a myriad of pathologies, including fifth disease in children, transient aplastic crisis, persistent anemia in immune-compromised patients, hydrops fetalis in pregnant women, and arthropathy [3–7]. B19V infects human erythroid progenitor cells (EPCs) through initial attachment to its primary receptor (P-antigen) [8] and interaction with co-receptors, resulting in virus internalization [9,10]. Virus replication and assembly take place in the nuclei of infected cells. The B19V double-stranded (ds) DNA replicative form (RF) genome expresses the large non-structural NS1 protein, two small non-structural proteins (the 11-kDa and 7.5-kDa proteins), and two capsid proteins (VP1 and VP2) [11–13].

B19V infects human EPCs during the late stages of maturation, particularly burst forming unit-erythroid (BFU-E) cells and colony forming unit-erythroid (CFU-E) cells [14–17]. B19V also infects non-erythroid tissues [18–20], but the infection of these tissues is non-productive, as virus replication is not fully supported [19,21,22]. Erythropoietin (EPO), a hormone secreted by renal tissue in response to hypoxia, is essential for survival, differentiation, and development of EPCs during the late maturation stages [23]. In addition to the role in survivability of EPCs, EPO/EPO receptor (EPO-R) signaling is essential to B19V replication [24]. EPO binding to EPO-R activates Janus kinase 2 (JAK2)-signal transducer and activator of transcription 5 (STAT5), phosphoinositide 3-kinase (PI3K), and extracellular signal-regulated kinase (ERK) pathways. The JAK2-STAT5 pathway positively regulates B19V replication, the ERK pathway negatively regulates B19V replication, and the PI3K pathway is dispensable to B19V replication [25]. Expression of STAT5A is upregulated during hypoxia, and replication of B19V in human EPCs is facilitated by hypoxic conditions [25]. JAK2 predominantly phosphorylates STAT5A in the cells of erythroid lineage [26], and thus STAT5A is largely involved in facilitating B19V replication of EPCs under hypoxic conditions [25]. The disease outcomes of hematological disorders caused by B19V infections result from the death of infected human EPCs. B19V infection inhibits erythropoiesis by inducing cell-cycle arrest [27–29], and eventually results in apoptosis [30–33].

The results of this study confirmed that phosphorylation of STAT5 is essential for B19V DNA replication. Mechanistically, the B19V RF DNA genome harbors STAT5-binding element (STAT5BE) within the minimal origins of DNA replication (*Ori*), located to the ITRs at each end of the viral genome. The binding site specifically binds phosphorylated STAT5 (pSTAT5). Moreover, our experiments revealed a novel interaction between STAT5 and minichromosome maintenance (MCM) complex; B19V exploits this interaction to recruit MCM complex to the viral replication centers for initiation of B19V DNA replication.

## Results

### Inhibition of STAT5 phosphorylation completely inhibited B19V DNA replication

**Fig 1:**
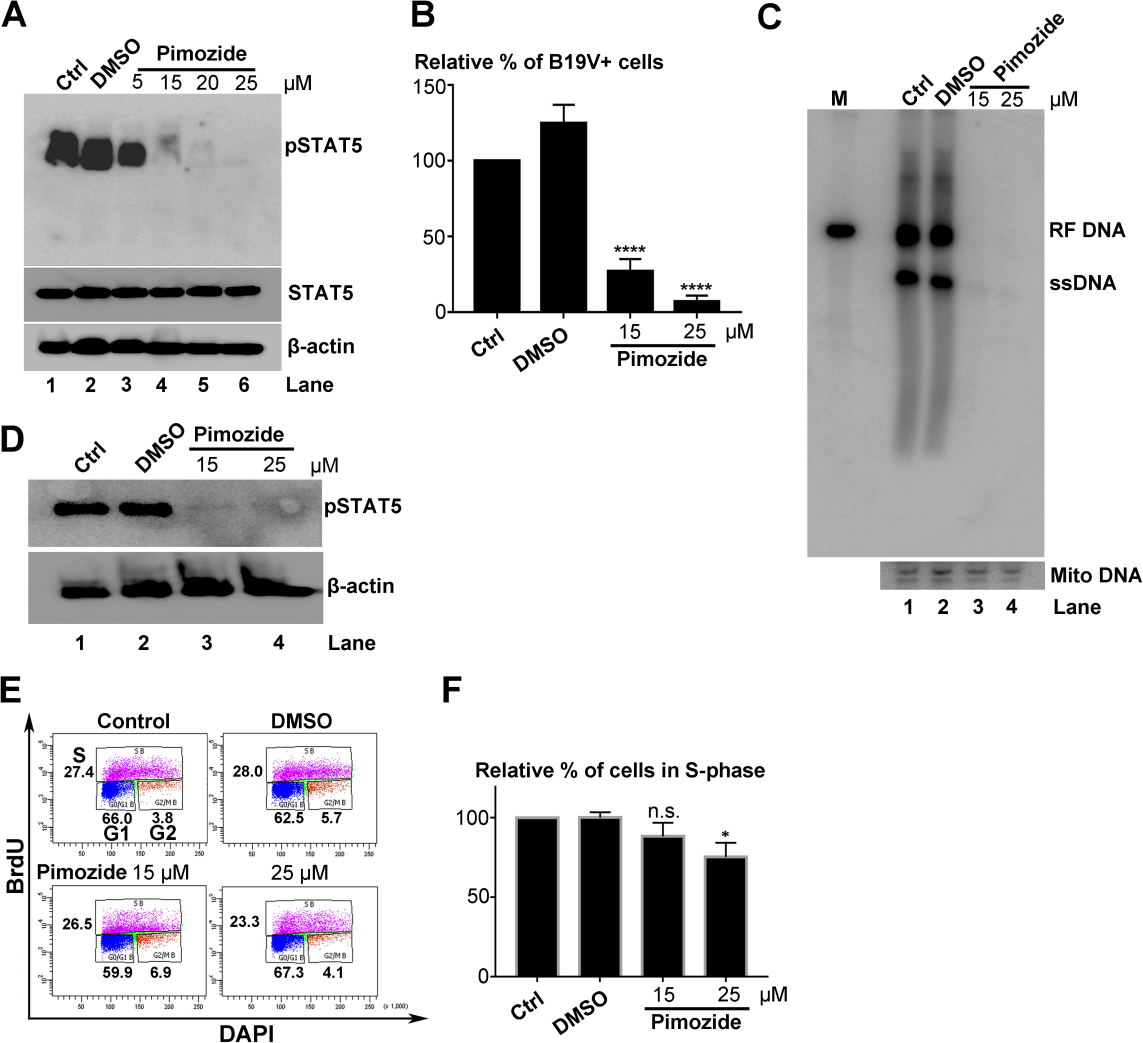
Pimozide abolishes B19V replication in primary CD36^+^ erythroid progenitor cells (EPCs). (A) Inhibition of phosphorylation of STAT5 by pimozide. CD36^+^ EPCs were treated with dimethyl sulfoxide (DMSO) vehicle or pimozide at various concentrations. At 48 h post-treatment, cells were collected, washed and lysed for Western blotting with detection of pSTAT5 by anti-pSTAT5(Y694) antibody. The blot was reprobed for total STAT5 using a mouse anti-STAT5 antibody and for β-actin as a loading control. (B-D) Inhibition of B19V DNA replication by pimozide. CD36^+^ EPCs were pre-incubated with DMSO or pimozide at a final concentration of 15 μM or 25 μM 6 h prior to B19V infection. At 48 h post-infection, cells were subjected to (B) flow cytometry analysis for the B19V-infected cell population with an anti-B19V capsid antibody, (C) Hirt DNA extraction, followed by Southern blot analysis with a B19V M20 DNA probe, or (D) Western blotting with an anti-pSTAT5(Y694) antibody, and reprobing with an anti-β-actin antibody. (E&F) Evaluation of the effect of pimozide on cell proliferation. CD36^+^ EPCs were treated with either DMSO or pimozide (15 μM or 25 μM). After 48 h, treated cells were incubated with bromodeoxyuridine (BrdU) for 1 h to analyze cell-cycle progression by a BrdU incorporation assay. (E) Results of a representative cell-cycle analysis experiment. (F) Relative fold change in the S phase cell population of each group is shown, with means and standard deviations of three independent experiments. P values are calculated using one-way and Tukey-Kramer post-test, compared with DMSO control. * denotes P<0.05; **** denotes P<0.0001; and n.s. (P>0.05) denotes no statistical significance.

Our results previously demonstrated that pSTAT5A has a critical role in B19V infection of human EPCs cultured under hypoxic conditions [25], leading us to consider in this study whether specific inhibition of STAT5 phosphorylation affects B19V replication. This possibility was tested by treating cells with a specific inhibitor of STAT5 phosphorylation, pimozide [34]. At a final concentration of 15 μM, pimozide abolished >90% of the STAT5 phosphorylation in CD36^+^ EPCs, without altering the total expression of STAT5 (**Fig 1A**, lane 4). CD36^+^ EPCs were incubated with pimozide 6 h prior to infection, and, at 48 h post-infection, numbers of B19V-infected (capsid-expressing) cells were reduced by 4.7-fold and 18.5-fold at 15 μM and 25 μM pimozide, respectively, compared with DMSO-treated cells (**Fig 1B**). Pimozide abolished viral DNA replication at both concentrations (**Fig 1C**). STAT5 dephosphorylation was confirmed in pimozide-applied infected cells (**Fig 1D**). These results suggested that inhibition of STAT5 phosphorylation abolishes viral DNA replication in B19V-infected CD36^+^ EPCs. Notably, treatment with pimozide at 15 μM did not significantly inhibit cell proliferation, as assessed by the BrdU incorporation assay (**Fig 1E & 1F**).

Pimozide treatment, at a concentration as low as 10 μM, also abolished DNA replication of the B19V RF genome M20 in transfected UT7/Epo-S1 cells (**S1A Fig**, lane 3), and inhibited STAT5 phosphorylation (**S1B Fig**, lane 3). As controls, at 10 or 20 μM pimozide, cell proliferation was not significantly affected (**S1C & S1D Fig**). Taken together, our results suggested that phosphorylation of STAT5 is essential for viral DNA replication.

### Phosphorylated STAT5 interacts with a consensus STAT5-binding element in the B19V minimal replication origin (*Ori*)

**Fig 2:**
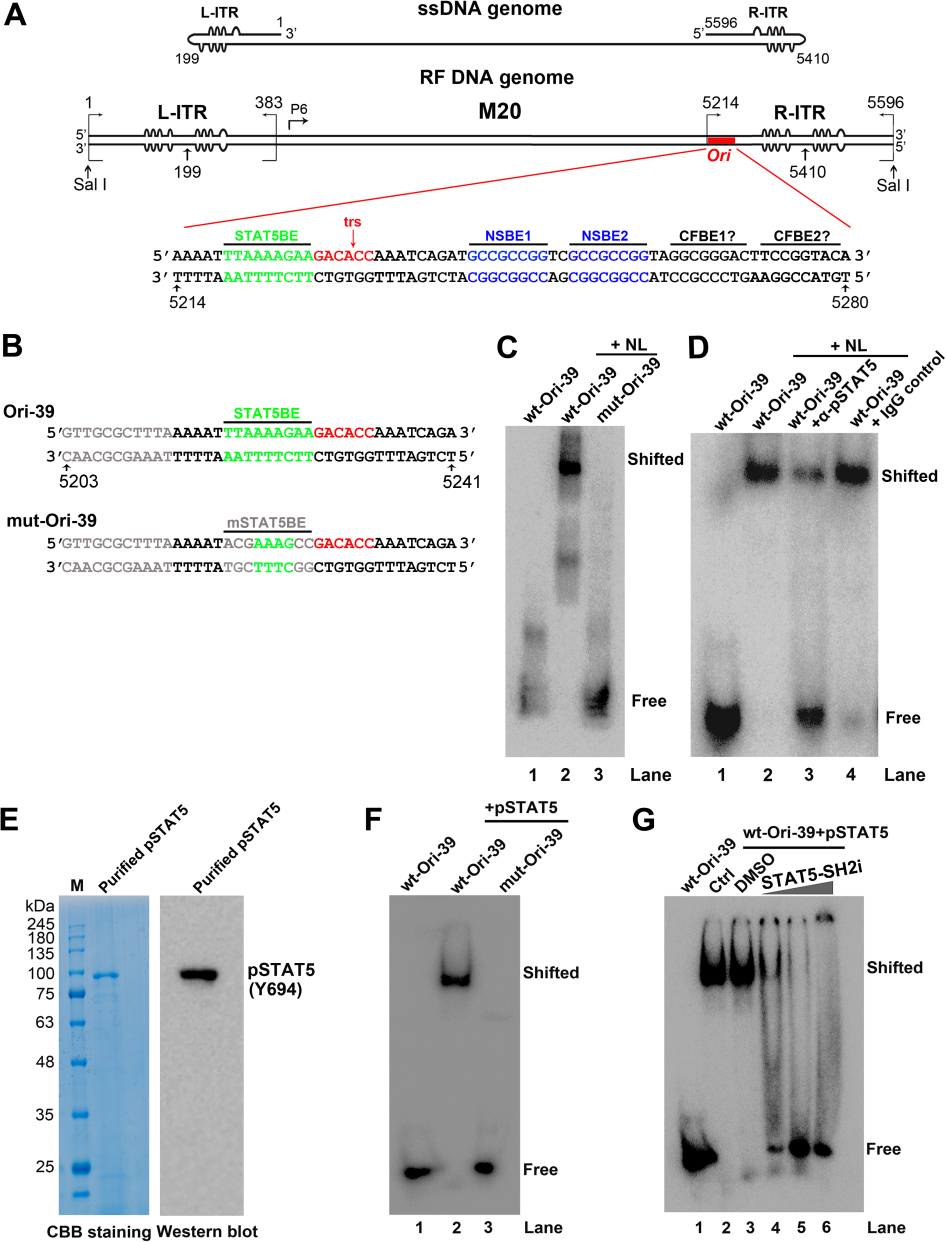
STAT5 interacts with B19V replication origins (*Ori*) *in vitro*. (A) Diagram of the B19V ssDNA and replicative form (RF) DNA genome. B19V genomes of the single-stranded (ss) DNA form and full-length replicative form (RF) are depicted, along with the sequence of viral *Ori* that contains a consensus STAT5-binding element (STAT5BE), terminal resolution site (trs), two NS1-binding elements (NSBE1 and NSBE2), and two putative cellular factor-binding elements (CFBE) [38–40]. (B) Probes used in electrophoretic mobility shift assay (EMSA). Sequences of two 39-nt probes, wt-Ori-39 and mut-Ori-39, are shown with the consensus STAT5BE and the mutated STAT5BE (mSTAT5BE) highlighted. (C&D) EMSA. (C) ^32^P-labeled *Ori* probes wt-Ori-39 (lane 2) and mut-Ori-39 (lane 3) were incubated with UT7/Epo-S1 nuclear lysate (NL) in the presence of non-specific competitor poly dI-dC. Products were subjected to non-denaturing 5% polyacrylamide gel electrophoresis (PAGE). Gels were dried and exposed to a phosphor screen. (D) Similarly, EMSA was performed with ^32^P-labeled wt*-Ori* probes and 5 μg of NL in the presence of 5 μg of anti-pSTAT5(Y694) or IgG control antibody. (E) PAGE analysis of purified pSTAT5. 20 μl of pSTAT5 was analyzed by SDS-10% PAGE. Gels were either stained with Coomassie brilliant blue (left panel/CBB staining), or transferred to a PVDF membrane for Western blotting with an anti-pSTAT5(Y694) antibody (right panel/Western blot). (F&G) EMSA with purified pSTAT5. (F) ^32^P-labeled wt-Ori-39 (lane 2) and mut-Ori-39 (lane 3) probes were incubated with purified pSTAT5 in the presence of poly dI-dC. Samples were run on 5% non-denaturing PAGE, dried, and exposed to a phosphor screen. (G) EMSA with wt-Ori-39 in the absence (lanes 2&3) or presence of STAT5-SH2i at 0.3 mM (lane 4), 0.5 mM (lane 5), and 0.8 mM (lane 6). Lane 1, wt-Ori-39 probe only.

The requirement of pSTAT5 for B19V DNA replication suggested that there might be a direct involvement of pSTAT5 in viral DNA replication. *In silico* analysis of the B19V genome demonstrated the presence of several consensus STAT5-binding elements (STAT5BEs) throughout the genome. STAT transcription factor binds a GAS or GAS-like motif with a consensus sequence of TTCN3GAA, TTCN3TAA, or TTAN3GAA [35]. TTCN3TAA binds STAT5 [36] and is one of the top ten STAT5BEs identified in a genome wide analysis by ChIP-seq [37]. A consensus STAT5BE is located within the previously identified 67-nt *Ori* in the B19V genome (**Fig 2A**) [38].

Binding of pSTAT5 from nuclear lysates of UT7/Epo-S1 cells to the STAT5BE in the *Ori* was confirmed by EMSA. A shifted band, indicating binding of protein to the probe, was observed in the presence of wild-type (wt) *Ori*-derived probe wt-Ori-39, but not the mut-Ori-39 that has the STAT5BE mutated (**Fig 2B and 2C**, lanes 2 vs 3). On incubation with an anti-pSTAT5 antibody, the level of shifted band was dramatically decreased (**Fig 2D**, lane 3). Because the EMSA was performed in the presence of excess amounts of non-specific competitor poly dI-dC, these results indicated specific binding of pSTAT5 to the B19V *Ori*.

pSTAT5 was purified from UT7/Epo-S1 cells by the use of beads conjugated with high affinity STAT5-binding DNA oligonucleotides (**Fig 2E**). EMSA was repeated with the purified pSTAT5, which shifted the labeled wt-Ori-39, but not the mut-Ori-39 (**Fig 2F**, lanes 2 vs 3). Shifting of wt-Ori-39 was abolished by addition of the STAT5-SH2 inhibitor, STAT5-SH2i, in a dose-dependent manner (**Fig 2G**, lanes 4–6). These binding assays confirm that pSTAT5 specifically binds to the STAT5BE of B19V *Ori in vitro*.

### Phosphorylated STAT5 is associated with replicating viral DNA in the viral DNA replication centers of B19V-infected EPCs

**Fig 3:**
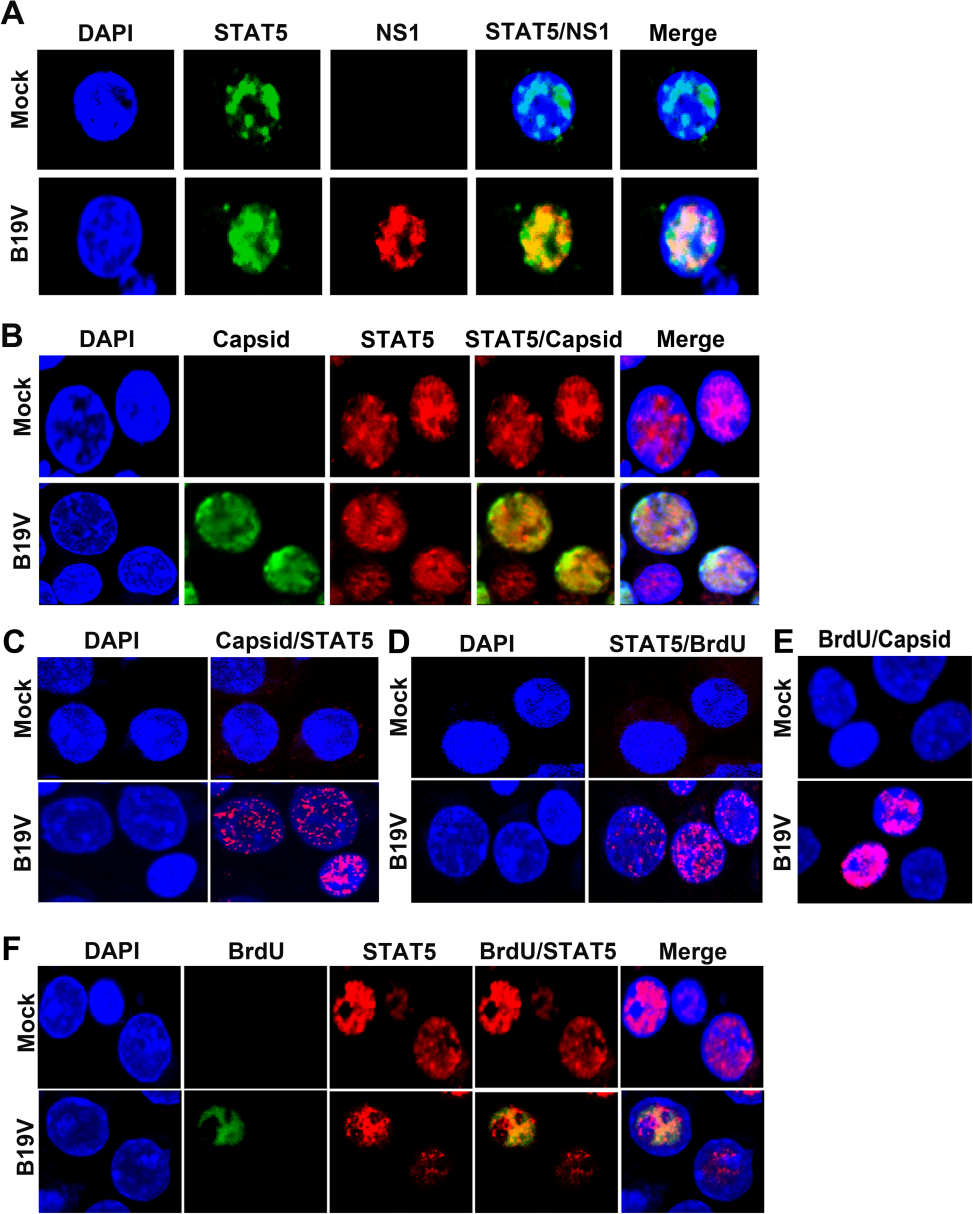
STAT5 colocalizes with B19V NS1, capsids, and the replicating B19V genome. (A&B) STAT5 colocalizes with B19V NS1 and capsids. Mock- or B19V-infected CD36^+^ EPCs were co-stained and examined with rabbit anti-STAT5 and rat anti-B19V NS1 antibodies (A) or with rabbit anti-STAT5 and mouse anti-B19V capsid antibodies (B). (C-E) Proximity ligation assay. Infected cells were co-stained with rabbit anti-STAT5 and mouse anti-B19V capsid antibodies (C), or co-stained with rabbit anti-STAT5 and mouse anti-BrdU antibodies (D), or co-stained with mouse anti-B19V capsid and rabbit anti-BrdU antibodies (E), followed by a proximity ligation assay, which produces amplified signal for labeled molecules in close proximity. (F) STAT5 colocalizes with the replicating viral genome. Mock- or B19V-infected CD36^+^ EPCs were BrdU labeled to identify replicating viral ssDNA genomes. The treated cells were co-stained with rabbit anti-STAT5 and mouse anti-BrdU antibodies, followed by incubation with secondary antibodies. Images were taken with an Eclipse C1 Plus (Nikon) confocal microscope at 100 × magnification. Nuclei were stained with DAPI.

The association of STAT5 with B19V NS1 and the viral capsid was demonstrated by immunofluorescence assays (**Fig 3A & 3B**). STAT5 colocalized with NS1 and the viral capsid in the nucleus of B19V-infected CD36^+^ EPCs. The association of STAT5 with viral capsid was confirmed by the observation of fluorescent foci in B19V-infected cells in a proximity ligation assay (**Fig 3C**), which produces an amplified signal when two labeled molecules are within 20 nm of one another [41].

**Fig 4:**
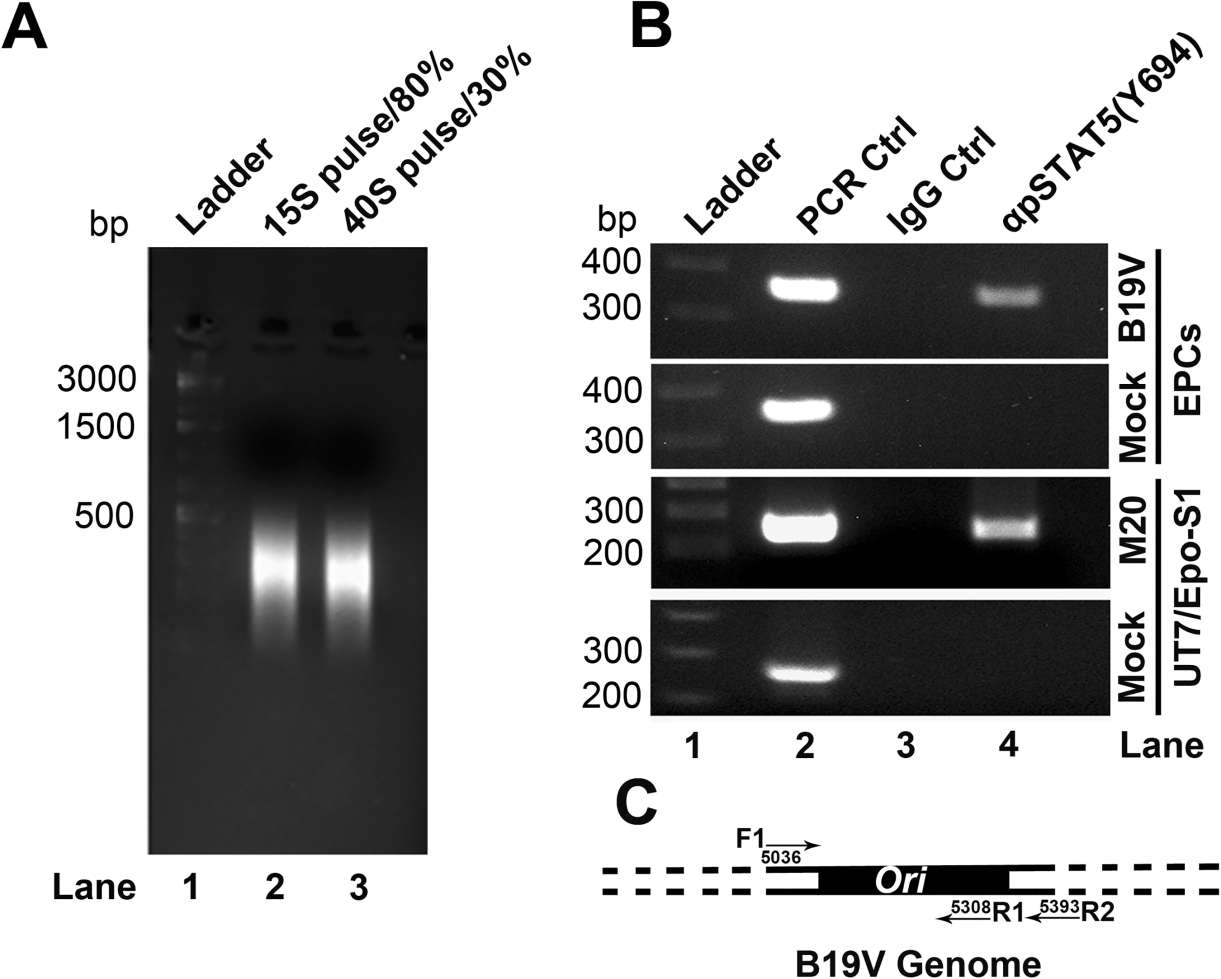
Chromatin immunoprecipitation (ChIP) assay. ChIP assay was performed using either infected CD36^+^ EPCs or transfected UT7/Epo-S1 cells, as indicated. (A) Crosslinked chromatin was sheared by sonication to sizes of ~500 bp. (B) An anti-pSTAT5(Y694) antibody or negative control IgG was used to pull down DNA-protein complexes. Recovered DNA from UT7/Epo-S1 cells or CD36^+^ EPCs was examined for viral DNA by PCR with primer sets of F1/R1 and F1/R2, respectively, which span the *Ori* sequences of the B19V genome. pM20 plasmid was used as a template for positive controls of PCR. (C) A diagram of the *Ori*-targeting PCR. The primers used for PCR are shown.

Proximity ligation assay (**Fig 3D**) and confocal microscopy (**Fig 3F**) both demonstrated that STAT5 colocalized with replicating viral DNA that was pulse-labelled with BrdU in B19V-infected CD36^+^ EPCs, which are parvovirus replication centers [42,43] as shown by proximity ligation assay using anti-BrdU and anti-capsid antibodies (**Fig 3E**). Interaction of pSTAT5 with the viral genome in cells was confirmed by ChIP assays in B19V-infected CD36^+^ EPCs and M20-transfected UT7/Epo-S1 cells. The pSTAT5-DNA complexes were pulled down with anti-pSTAT5(Y694) antibody, and bound viral *Ori* was detected by PCR. In the ChIP assay, cellular DNA was sheared to < 500 bp by sonication (**Fig 4A**). A specific PCR band was amplified in samples from B19V-infected or M20-transfected cells pulled down by anti-pSTAT5(Y694) (**Fig 4B**, lane 4). Moreover, in UT7/Epo-S1 cells transfected with the B19V RF genome (M20), we observed that application of pimozide significantly decreased the amount of the *Ori*-containing fragments of the M20, as assessed by the quantitative ChIP assay targeting *Ori* (**S4C Fig**, Pimozide). Thus, these results confirmed the association of pSTAT5 with B19V *Ori* in B19V-infected CD36^+^ EPCs and M20-transfected UT7/Epo-S1 cells.

### Disruption of the interaction between STAT5 and viral *Ori* inhibits viral DNA replication

**Fig 5:**
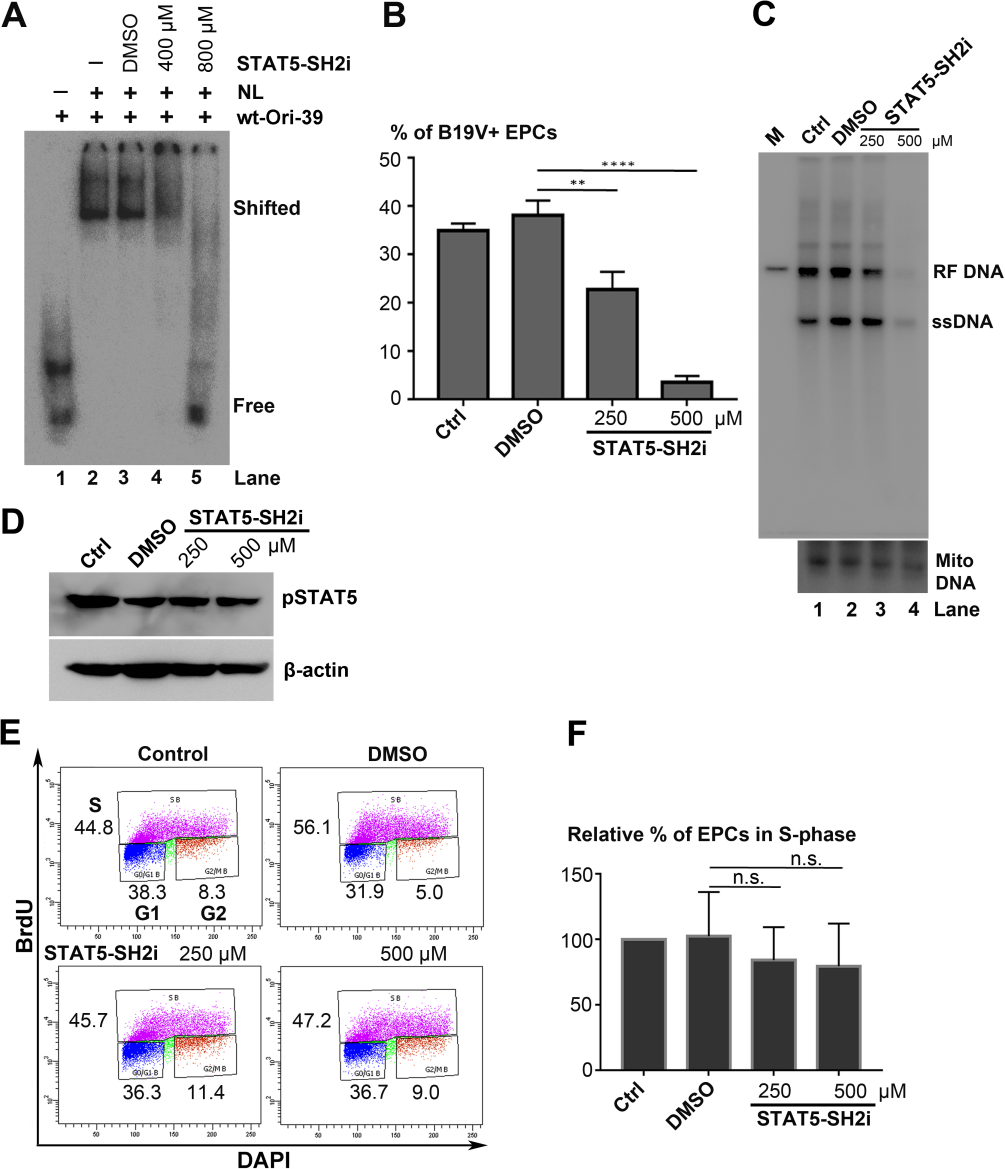
Blockage of interaction between STAT5 and B19V *Ori* DNA inhibits B19V replication. (A) STAT5-SH2 inhibitor (STAT5-SH2i) abolishes the shift of viral *Ori* in EMSA. UT7/Epo-S1 nuclear lysate (NL) was incubated with ^32^P-labelled wt-Ori-39 probe (lanes 2–5) with the addition of DMSO (lane 3) or STAT5-SH2i at 0.4 mM (lane 4) and 0.8 mM (lane 5). (B-D) STAT5-SH2i significantly inhibits viral DNA replication. CD36^+^ EPCs were incubated with either DMSO or STAT5-SH2i (250 μM or 500 μM), 6 h prior to infection. At 48 h post-infection, cells were collected and subjected either to (B) flow cytometry analysis for the B19V-infected (B19V+) cell population, with an anti-capsid antibody, or to (C) Hirt DNA extraction for Southern blot analysis with a B19V M20 DNA probe (upper panel), with mitochondrial DNA (Mito DNA) probed as a loading control (lower panel), or to (D) protein extraction for Western blotting with anti-pSTAT5 and anti-β-actin (E&F) Effect of STAT5-SH2i on cell proliferation. CD36^+^ EPCs were treated with either DMSO or STAT5-SH2i (250 μM or 500 μM), and were then incubated with BrdU to perform BrdU incorporation assays. (E) Results of a representative cell-cycle analysis. (F) Relative fold changes in the S-phase cell population of each group shown with means and standard deviations of three independent experiments. P values are calculated using one-way ANOVA and Tukey-Kramer post-test (P>0.05), compared with DMSO control. **** denotes P<0.0001, ** P<0.01, and n.s. no statistical significance.

A small molecule STAT5-SH2 inhibitor (STAT5-SH2i, CAS no. 285986-31-4) specifically targets the SH2 domain of STAT5 and inhibits STAT5 binding to DNA [44]. EMSA was performed to determine whether STAT5-SH2i disrupts the interaction between the STAT5 and B19V *Or*i. Incubation of either UT7/Epo-S1 nuclear lysates or purified pSTAT5 with increasing concentrations of the inhibitor showed that the STAT5-SH2i prevented formation of the STAT5-DNA formation in a dose-dependent manner (**Fig 5A and Fig 2G**). To examine the effect of the inhibitor on virus replication, CD36^+^ EPCs were pretreated with STAT5-SH2i 6 h prior to infection with B19V. The results showed that, at a final concentration of 500 μM, the inhibitor significantly decreased the virus-infected cell population by 10.7-fold (**Fig 5B**), and the level of viral RF DNA by ~10-fold (**Fig 5C**), but not the expression level of pSTAT5 (**Fig 5D**), compared with the cells with DMSO treatment. Cell proliferation was not significantly affected by this level of inhibitor in mock-infected CD36^+^ EPCs (**Fig 5E & 5F**). The inhibition of viral DNA replication by STAT5-SH2i was also demonstrated in M20-transfected UT7/Epo-S1 cells (**S2 Fig**), and STAT5-SH2i significantly disrupted the interaction of pSTAT5 with the *Ori* of the B19V RF genome (M20) *in vivo* as shown by a ChIP assay (**S4C Fig**, STAT5-SH2i).

### Derivatives of B19V replicative form genome with mutated STAT5-binding elements do not replicate in UT7/Epo-S1 cells

**Fig 6:**
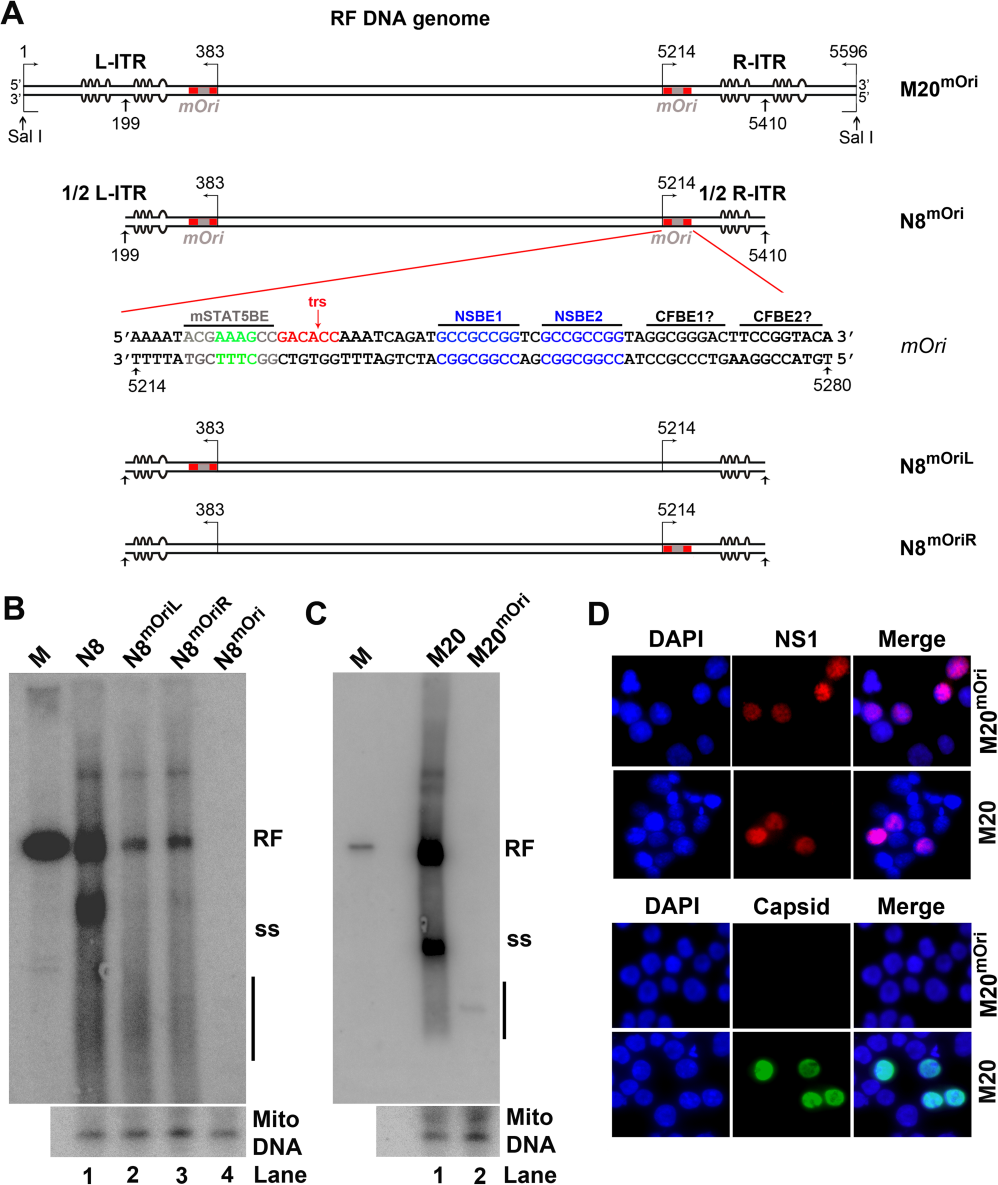
Failure of B19V replicative form DNA clones with STAT5-binding element mutations in replication in transfected UT7/Epo-S1 cells. (A) Diagram of the B19V full-length M20 RF genome and various half ITR-deleted N8 RF genomes with mutations at STAT5-binding elements (STAT5BEs). Red squares indicate the position of the *Ori* sequences at both ITRs, and grey squares indicate mutated Ori (*mOri*). The sequence of the *mOri* is shown with mutated nucleotides in grey in the STAT5BE. (B&C) Southern blot analysis. (B) The N8 RF DNA, or derivatives with mutations in the STAT5BE of either the left ITR (N8^mOriL^), right ITR (N8^mOriR^), or both (N8^mOri^), were transfected into UT7/Epo-S1 cells. (C) M20, and M20^mOri^, a derivative of the M20 RF DNA with STAT5BEs of both ITRs mutated, were transfected into UT7/Epo-S1 cells. At 48 h post-transfection, cells were collected for Hirt DNA extraction. And Hirt DNA samples were analyzed by Southern blotting with an M20 DNA probe. RF DNA (RF), ssDNA (ss), and Dpn I-digested DNA (shown with a line) are indicated. Mitochondrial DNA (Mito DNA) was used as a loading control (lower panels). (D) Viral protein expression of B19V DNA mutants. M20 or M20^mOri^ transfected UT7/Epo-S1 cells were stained with anti-NS1 or anti-capsid antibodies. Confocal images were taken with an Eclipse C1 Plus (Nikon) microscope at 100 × magnification.

The effect of mutation of the STAT5BE of the viral *Ori* on replication of the B19V RF genome was determined. The viral genome has an *Ori* sequence adjacent to each ITR, and the STAT5BE was mutated in either the left ITR (N8^mOriL^) or right ITR (N8^mOriR^) or both ITRs (N8^mOri^) of the N8 replicating RF DNA that has half ITRs at both ends, as shown in **Fig 6A**. The replication capability of these mutated RF genomes was examined in UT7/Epo-S1 cells. Although the N8 RF DNA replicated well, much less replication occurred with N8^mOriL^ and N8^mOriR^, and no replication was observed with N8^mOri^ (**Fig 6B**). The mutations in the STAT5BEs were then introduced into both ITRs of M20 RF genome, to make the M20^mOri^ mutant. No viral DNA replication was observed in M20^mOri^-transfected cells (**Fig 6C**, lane 2). Although both M20 and M20^mOri^ RF genomes expressed NS1, viral capsid (a hallmark of B19V DNA replication [45]) was present only in M20-transfected cells (**Fig 6D**).

### pSTAT5 interacts with the MCM complex of the pre-initiation complex of cellular DNA replication

During initiation of cellular DNA replication, the origin recognition complex (ORC) binds to autonomously replicating sequence sites and recruits cell division control protein (CDC6) and DNA replication factor CDT1 to replication origins [46]. CDT1 recruits the MCM complex and primes replication initiation [47]. Although viral DNA replicates independently of ORC/CDC6/CDT1, DNA viruses may require the MCM complex to initiate viral DNA replication [48]. In the parvovirus adeno-associated virus (AAV), MCM complex is required for *in vitro* reconstitution of viral DNA replication [49]. In the case of B19V, we previously found that MCM complex is associated with the viral DNA replication centers and has a role in B19V replication [50].

**Fig 7:**
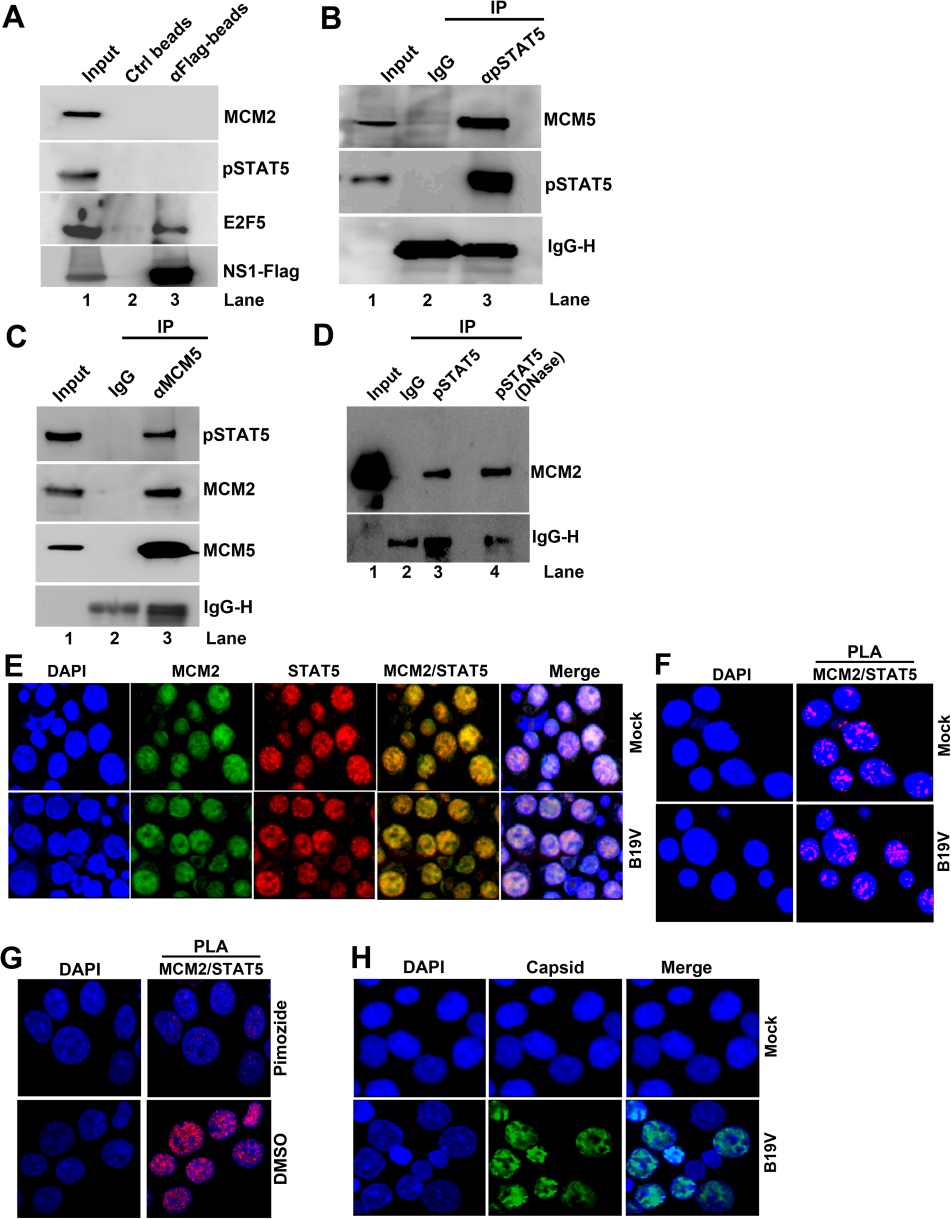
pSTAT5, but not NS1, interacts with the MCM complex. (A) Immunoprecipitation (IP) assay. Cell lysates of NS1^Flag^-expressing UT7/Epo-S1 cells were prepared for pull-down assays with either anti-Flag-conjugated beads or control beads. Immunoprecipitated proteins were examined for the presence of MCM2 by Western blotting. Blots were reprobed with rabbit anti-pSTAT5(Y694), anti-E2F5, and anti-Flag antibodies. Detection of E2F5 was used as a positive control for NS1 IP. (B) Co-IP assay. UT7/Epo-S1 cells were collected, washed, and lysed with RIPA buffer. After centrifugation, the supernatant was incubated with either rabbit anti-pSTAT5(Y694) or control IgG antibody. Immunoprecipitated proteins were blotted for the presence of the MCM complex with an anti-MCM5 antibody and for pSTAT5 with rabbit anti-pSTAT5(Y694). (C) Reverse Co-IP assay. Reverse Co-IP was performed with an anti-MCM5 antibody. Immunoprecipitated proteins were examined for pSTAT5, MCM2, and MCM5, respectively. (D) Co-IP of lysates treated with DNase. UT7/Epo-S1 cell lysates, either treated or untreated with DNase (750 units of Benzonase) were incubated with anti-pSTAT5(Y694) or control IgG antibodies for Co-IP assay, and immunoprecipitated proteins were examined for MCM2 by Western blot analysis. (E-H) Immunofluorescence analysis. (E&F) Mock- or B19V-infected CD36^+^ EPCs were co-stained with rabbit anti-STAT5 and mouse anti-MCM2 antibodies, followed by (E) incubation with respective secondary antibodies, or by (F) proximal ligation assay, which produces amplified signal for labeled molecules in close proximity. (G) CD36^+^ EPCs were incubated with either DMSO or pimozide (at 30 μM) for 2 days. And then the cells were co-stained with rabbit anti-STAT5 and mouse anti-MCM2 antibodies for proximity ligation assay. (H) Infected EPCs were stained with an anti-capsid antibody. Confocal images were taken with an Eclipse C1 Plus (Nikon) microscope at 100 × magnification.

Initially, to determine whether the viral NS1 protein has a role in recruitment of the MCM complex to the viral replication origin, we performed pull-down assays using lysates from NS1-expressing UT7/Epo-S1 cells. With pull-down of NS1, MCM and pSTAT5 were not detected (**Fig 7A**, lane 3), but the positive control transcription factor E2F5 (which interacts with B19V NS1 [27]) was detected, which suggested that NS1 has no role in recruitment of the MCM complex. By contrast, co-immunoprecipitation (Co-IP) with an anti-pSTAT5 antibody pulled down MCM5 protein of the MCM complex from lysates of UT7/Epo-S1 cells (**Fig 7B**). Similarly, Co-IP with an anti-MCM5 antibody pulled down pSTAT5, in addition to MCM2 (**Fig 7C**). The interaction between pSTAT5 and the MCM complex was DNA-independent, as DNase treatment of the lysate did not disrupt the interaction (**Fig 7D**, lane 4). Also, we show that MCM2, MCM3, MCM5 and MCM7 were associated with viral *Ori* in M20-transfected UT7/Epo-S1 cells, as confirmed by ChIP analyses **(S4A Fig).**

STAT5 and the MCM complex colocalized in CD36^+^ EPCs, irrespective of whether the cells were infected (**Fig 7E**). An association of the MCM complex with STAT5 was confirmed in both B19V- and mock-infected cells by the proximity ligation assay (**Fig 7F**). This association was blocked by treatment of pimozide in CD36^+^ EPCs (**Fig 7G**). Immunofluorescence detection of the viral capsid demonstrated that, following B19V infection, most of the cells were infected **(Fig 7H).**

### pSTAT5 recruits MCM complex to B19V *Ori* to facilitate initiation of viral DNA replication

**Fig 8:**
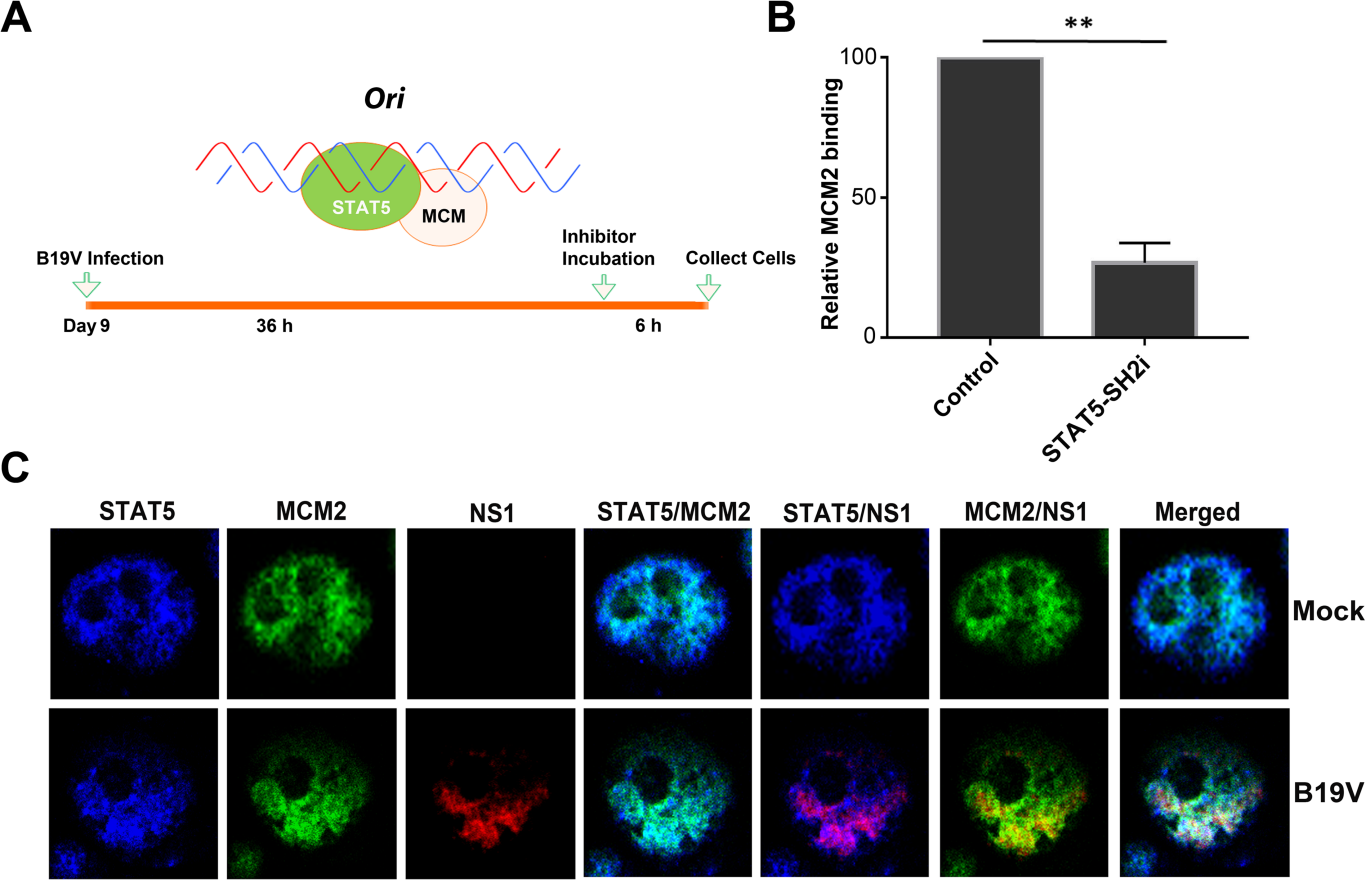
The MCM complex is loaded onto the viral *Ori*. (A) Experimental strategy. The STAT5-MCM complex is depicted interacting with a DNA sequence, such as the viral *Ori*. The time line of B19V infection and treatment with the STAT5-SH2 inhibitor (STAT5-SH2i) shows time (h) post-infection or post-treatment. (B) ChIP assay. Cells were treated as shown in panel A. An anti-MCM2 antibody was used to pull down the STAT5-MCM-DNA complex, and the recovered ChIP DNA was subjected to qPCR with a primer set spanning the *Ori* region. Compared with the absence of the STAT5-SH2i, the relative abundance (percentage) of MCM on the viral *Ori* in the presence of the STAT5-SH2i is shown, with mean and standard deviation of three independent experiments. P values are calculated using a Student’s t test, ** denotes P<0.01. (C) Three color confocal microscopy. Mock- or B19V-infected CD36^+^ EPCs were co-stained with rabbit anti-STAT5, rat anti-NS1, and mouse anti-MCM2 antibodies, followed by staining with secondary antibodies conjugated with Dylight405, Texas Red, and FITC, respectively. Images were taken with an Eclipse C1 Plus (Nikon) confocal microscope at 100 × magnification.

Our demonstration that pSTAT5 interacts with viral *Ori* as well as the MCM complex suggested that B19V might exploit these interactions to initiate viral DNA replication. To test this hypothesis, we infected CD36^+^ EPCs with B19V, and at 36 h post-infection (when B19V DNA replication was at its peak), we treated the cells with STAT5-SH2i (**Fig 8A**). At 6 h post-treatment, the cells were collected for ChIP assay with anti-MCM2 antibody, which showed that MCM abundance on viral *Ori* decreased significantly in the presence of STAT5-SH2i, compared with untreated control cells (**Fig 8B**). Results with three-color confocal imaging demonstrated that MCM2 and STAT5 colocalized in mock-infected cells (in the absence of viral NS1) (**Fig 8C**, Mock). In infected cells, viral NS1 (which binds viral *Ori*) colocalized with both STAT5 and MCM, indicating that they were localized at viral DNA replication centers (**Fig 8C**, B19V). These results suggested that B19V utilizes viral *Ori*-STAT5 and STAT5-MCM interactions to recruit the MCM complex to viral DNA replication origins, to initiate viral DNA replication.

### Pimozide is a promising candidate for the treatment of B19V infection

**Fig 9:**
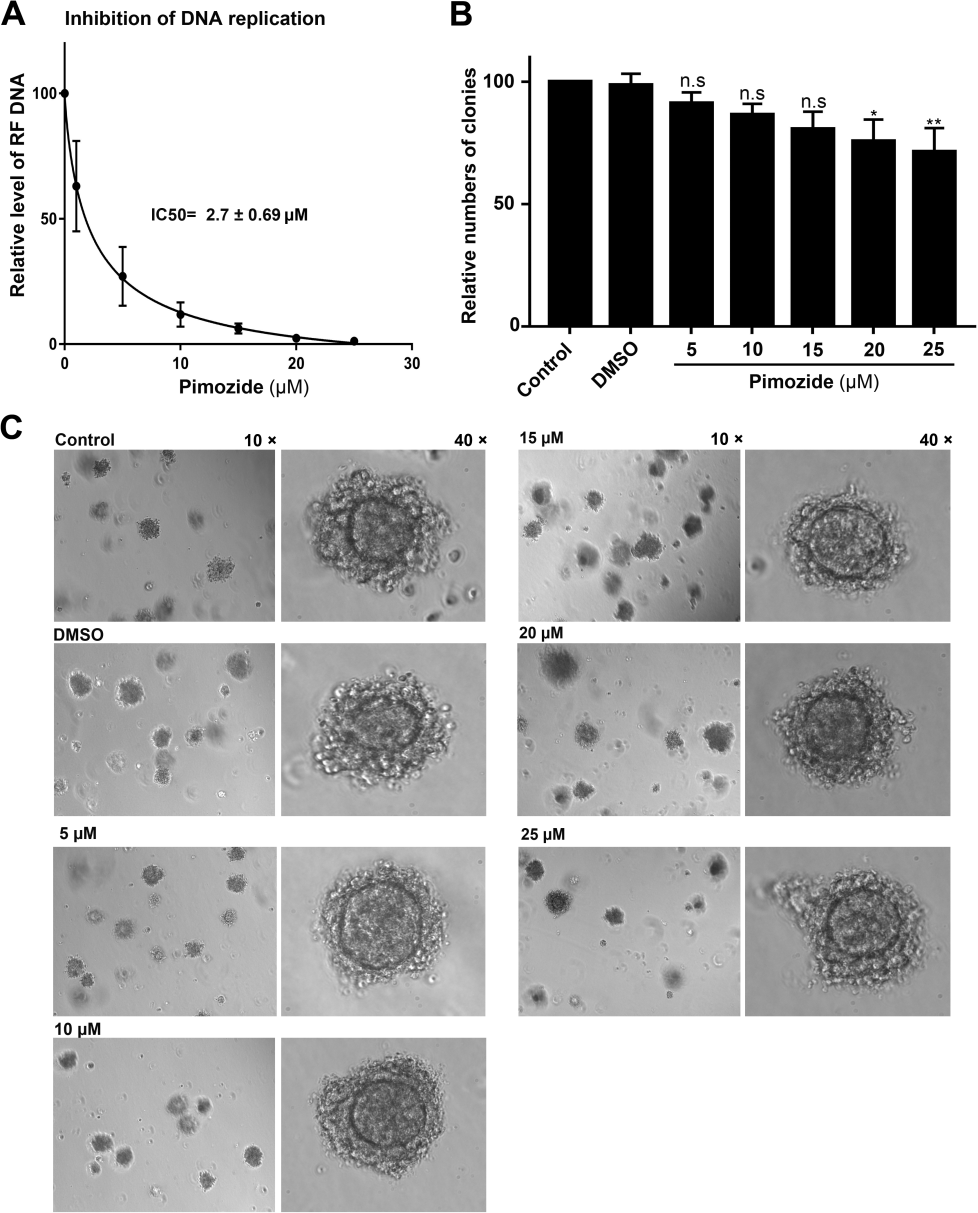
IC50 determination and colony formation assay. (A) IC_50_ determination. CD36^+^ EPCs incubated with pimozide at different concentrations were infected with B19V. After 48 h post-infection, the cells were extracted for Hirt DNA. The DNA samples were examined for B19V DNA with Southern blot analysis using a M20 RF DNA probe. Viral RF DNA was quantified. RF DNA levels with pimozide relative to levels without pimozide are plotted against the concentrations of pimozide for the calculation of the IC_50_ value with GraphPad Prism. (B&C) Colony formation assay. CD36^+^ EPCs were incubated with pimozide at different concentrations for 48 h, and then cultured in methyl cellulose-containing medium. After 10 days, numbers of the colonies were counted (B). P values are calculated using one-way ANOVA followed by Tukey-Kramer post-test, compared with DMSO group. ** P<0.01; * P< 0.05; n.s. (P>0.05) denotes no statistical significance. Images of colonies were taken with Eclipse C1 Plus (Nikon) inverted microscope at 10 × and 40 × magnifications (C).

To confirm the efficacy of pimozide as a drug, we treated primary CD36^+^ EPCs with pimozide at various concentrations, and infected them with B19V. The cells were collected 48 h post-infection for quantification of viral DNA replication (RF DNA) by Southern blot analysis, which demonstrated that the IC_50_ of pimozide for inhibition of viral DNA replication (the concentration at which 50% of viral DNA replication was inhibited) was 2.7 ± 0.69 μM (mean ± standard error) (**Fig 9A**). To examine the effect of pimozide on colony formation in the absence of virus infection, CD36^+^ EPCs were incubated with pimozide at increasing concentrations on Day 7 for 2 days, and then cultured in methyl cellulose-based medium for colony formation. After 10 days, numbers of colonies were counted (**Fig 9B**). Pimozide only moderately reduced the numbers of colonies at higher concentrations (20–25 μM), but it did not affect the size or morphology of the colonies formed **(Fig 9C**).

## Discussion

We have now demonstrated that STAT5 is directly involved in B19V DNA replication. Importantly, STAT5 specifically interacts with the MCM complex, the eukaryotic DNA helicase complex that is required for the formation and elongation of the cellular DNA replication fork [51]. We therefore propose a novel model of B19V DNA replication in human EPCs, in which STAT5 functions as a mediator protein that brings the MCM complex to the viral DNA replication origins. Our results also identify pSTAT5 as a target for inhibition of B19V infection. In addition, as the STAT5-MCM interaction is independent of infection, we envisage an important role of this interaction in the context of cellular replication and transcription in human EPCs, which warrants further investigation.

### Phosphorylated STAT5A is directly involved in B19V replication

STAT5 is phosphorylated at a single conserved tyrosine residue (Tyr694 in STAT5A and Tyr699 in STAT5B), and these phosphotyrosine motifs, upon intermolecular interaction, enable formation of either homodimers or heterodimers of STAT5A/B [52,53]. These dimers accumulate in the nucleus and bind DNA, to transactivate target genes [52]. EPO-activated JAK2 phosphorylates STAT5 in human EPCs [54]. We examined the relative expression of STAT5A and STAT5B in UT7/Epo-S1 and CD36^+^ EPC lysates with a STAT5A/B pan-specific antibody, and found that STAT5A was predominantly expressed in both cell types (**S3A Fig**). This result agrees with the observations that JAK2 kinase predominantly phosphorylates STAT5A in cells of erythroid lineage [26], and a constitutively phosphorylated STAT5A (1*6) variant enhances virus replication, whereas knockdown of *STAT5A* inhibits virus replication in B19V-infected EPCs [25].

STAT5B promotes viral DNA replication, but, during replication of human papillomavirus 16 (HPV16), STAT5B enhances viral DNA replication indirectly via regulation of *TopBP1* expression, leading to the activation of ATR kinase [55]. In a proof-of-concept experiment, fusion of STAT5BEs to the DNA replication origin of polyoma virus replicon DNA improved replication efficiency in transfected mouse lymphoid BA/F3 cells, corroborating the direct role of STAT5 in viral DNA replication [56]. CD36^+^ EPCs have to be cultured in the presence of EPO for proliferation and differentiation [24], which dominantly leads activation of STAT5A (**S3A Fig**) through the EPO-JAK2-STAT5 pathway [25]; however, a DDR or activation of ATR is not observed in normal (uninfected) CD36^+^ EPCs [29,57] (**S5A Fig**). Furthermore, in hydroxyurea-treated CD36^+^ EPCs, both ATR and ATM were activated; however, application of pimozide did not change the level of phosphorylated ATR or ATM (**S5A Fig**). As ATR activation enhances B19V replication [57], these lines of evidence suggest that pSTAT5 does not utilize the STAT5-ATR pathway to facilitate B19V replication in CD36^+^ EPCs. Moreover, B19V infection *per se* did not affect STAT5 phosphorylation (**S5B Fig**). Of note, the binding of pSTAT5 to the *Ori*, which locates in front of the B19V P6 promoter, did not obviously transactivate the P6 promoter (**S6 Fig**). Thus, our results provide the first evidence that an authentic virus, B19V, depends on direct binding of pSTAT5 to its replication origin (*Ori*) for viral DNA replication.

### Phosphorylated STAT5 interacts with the MCM complex and recruits it to viral replication origins during DNA replication initiation

B19V infection induces late S-phase arrest in human EPCs, and S-phase factors are fully utilized by the virus to replicate its genome [50]. During cellular DNA replication, ORC-CDC6-CDT1 binding to the replication origin is a priming event that takes place in G1-phase [51]. Furthermore, CDT1 recruits the MCM complex and subsequently the whole replisome via formation of the MCM-CDC45 complex [51]. Notably, no such priming takes place during S-phase, so that chromosomes are not replicated multiple times [46]. However, viruses have evolved different mechanisms to initiate viral DNA replication. For examples, SV40 has the large T antigen that binds SV40 DNA replication origin and has helicase activity, and also recruits the replication machinery by interacting with DNA replication factors, such as replication factor A, DNA polymerase α and topoisomerase I [58]. Parvoviruses use the large non-structural protein NS1, which binds directly to the viral origin and has helicase and nickase activities that facilitate viral DNA replication [59]. In parvovirus AAV, the MCM complex is essential to AAV2 DNA replication *in vitro* [49], and is probably recruited by interaction with Rep78, the large viral non-structural protein [60].

In the case of B19V, the MCM complex is localized to the viral DNA replication centers and is required for viral DNA replication [50]. However, we did not observe any interaction between the B19V NS1 protein and the MCM complex, suggesting that the complex is recruited to the viral DNA replication centers by an alternative mechanism. Here, our results provided evidence that STAT5 interacts with the MCM complex in human EPCs, without involvement of viral or cellular DNA. These cells express STAT5A more abundantly than STAT5B (**S3A Fig**), but both STAT5A and STAT5B proteins interact with the MCM complex (**S3C Fig**).

During B19V infection, STAT5 is recruited to the viral DNA replication origin by direct interaction with STAT5BEs in the *Ori* sequences of the viral genome, thereby bringing the MCM complex to the viral *Ori*. Outside of the *Ori*, there are additional 6 putative STAT5BEs, and we tested that two of them in the capsid proteins-coding region also bound pSTAT5 (**S4D Fig**). Since there is no putative terminal resolution site (trs) and NS1-binding sites outside of the *Ori*, we speculate that these STAT5BEs outside of the *Ori* do not contribute to B19V DNA replication. We hypothesize that MCM complex recruited by pSTAT5 at *Ori* may contribute to virus replication through its helicase activity or the recruitment of other DNA replication factors to the viral origin [51]. Notably, PIF (parvovirus initiation factor), a member of the KDWK family of transcription factors, has been shown to bind two adjacent “ACGT” motifs in front of the NS1 binding site of left-hand replication origin (OriL_TC_) of the *Protoparvoviurs* minute virus of mice (MVM) [61,62]. PIF stabilizes the binding of NS1 to the *Ori*, which is critical for the activation of NS1 nickase [63]. In B19V, at least in an *in vitro* nicking assay, B19V NS1 is sufficient to cleave the *Ori* [40]. However, whether the binding of STAT5 to B19V *Ori* or the recruited MCM complex also involves in NS1 nickase activity of the *Ori* at trs (**Fig 2A**) warrants further investigation.

### Pimozide, an FDA-approved drug, shows promise for the treatment of B19V infection

To date, no specific treatment (either anti-viral or vaccine-based) exists for B19V infection. We have now demonstrated that pimozide, an FDA-approved anti-psychotic drug that is used in the treatment of a wide range of diseases [64] and could be potentially used to treat chronic myeloid leukemia, in which it specifically targets cancer cells, without affecting CD34^+^ hematopoietic stem cells [34]. Pimozide specifically inhibits STAT5 phosphorylation without affecting JAK2 activation or JAK2-derived signaling pathways; however, the underlying mechanism is unknown yet [65]. The pSTAT5 is presumably required for recruitment of the MCM complex to the viral *Ori*, and facilitates B19V replication in human EPCs. Pimozide is a potent inhibitor of B19V replication, with an IC_50_ of ~2.7 μM. At 15 μM, pimozide does not have a significant effect on proliferation of human EPCs expanded *ex vivo*, and has only moderate effect (~15% reduction) on colony formation of EPCs. As STAT5A phosphorylation plays a key in B19V replication in human EPCs under hypoxic conditions [25], these lines of evidence suggest that the inhibition of B19V replication in CD36^+^ EPCs is not a side-effect of the pimozide. Antivirals such as cidofovir and ribavirin are used in the treatment of adenovirus infection, and have IC_50_ values of 15 μM for cidofovir and 25 μM for ribavirin [66]. Importantly, when we applied both pimozide and STAT5-SH2i (at 15 and 250 μM, respectively), a significant synergistic inhibition of B19V infection was observed (**S7 Fig**). Therefore, we expect that a clinical trial should be conducted to examine pimozide as a treatment for B19V infection of patients with sickle-cell disease and immunocompromised patients and as anti-viral prophylaxis of transplant recipients.

## Materials and methods

### Ethics statement

We purchased CD34^+^ hematopoietic stem cells, which were isolated from bone marrow of a healthy human donor, from AllCells LLC (Alameda, CA) without any identification information on the cells, and, therefore, an institutional review board (IRB) review was waived.

### Primary cells and cell lines

Primary human CD36^+^ EPCs were expanded *ex vivo* from CD34^+^ hematopoietic stem cells as previously described [24,25,67]. Briefly, hematopoietic CD34^+^ stem cells, purchased from AllCells, LLC (Alameda, CA), were grown in Wong medium under normoxia up to Day 4 and frozen in liquid nitrogen [25]. In each experiment, Day 4 cells were thawed and grown under normoxia in an atmosphere containing 5% CO_2_ and 21% O_2_ at 37°C for 2–3 days, prior to incubation under hypoxia at 5% CO_2_ and 1% O_2_.

The megakaryoblastoid cell line, UT7/Epo-S1, was cultured in Dulbecco's modified Eagle’s medium with 10% fetal bovine serum and 2 U/ml of EPO (Amgen, Thousand Oaks, CA) in 5% CO_2_ and 21% O_2_ at 37°C [38,68]. A UT7/Epo-S1 cell line expressing B19V NS1 protein (NS1-S1) was cultured under the same conditions, except that 5 μg/ml doxycycline was used to induce NS1 expression when needed [29].

### Virus and infection

Plasma samples containing B19V at ~1 × 10^12^ viral genomic copies per ml (vgc/ml) were obtained from ViraCor Eurofins Laboratories (Lee’s Summit, MO). After 2 days of hypoxia, CD36^+^ EPCs were infected with B19V at a multiplicity of infection (MOI) of ~1,000 vgc per cell. At 48 h post-infection, the infected cells were analyzed.

### Chemical inhibitors

STAT5-SH2 Inhibitor (STAT5-SH2i, CAS 285986-31-4; catalog number (cat#) 573108), a cell-permeable compound that selectively targets the SH2 domain of STAT5 [44], and STAT5 Inhibitor III, pimozide (CAS 2062-78-4, cat# 573110), which dephosphorylates STAT5 [34], were purchased from EMD Millipore (Billerica, MA). Both chemicals were dissolved in DMSO to produce stock solutions (at 100mM) that were kept at -80°C.

### Proximity ligation assay

Duo link In-Situ Red Mouse/Rabbit kit (cat# DUO92101) was purchased from MilliporeSigma (St Louis, MO). Proximity ligation assay was performed following the manufacturer’s instructions, as described previously [69].

### Immunofluorescence assay and confocal imaging

Immunofluorescence assay was carried out as described previously [25,50]. Briefly, infected EPCs were deposited on slides by cytospinning, fixed with 3.7% paraformaldehyde for 30 min, and permeabilized with phosphate-buffered saline (PBS, pH7.2) containing 0.5% Triton X-100 (PBS-T) for 5 min at room temperature. Non-specific interactions were blocked with 3% bovine serum albumin (BSA) before subsequent incubation with primary and fluorescence-labelled secondary antibodies. The slides were visualized with a Nikon confocal microscope, and images were taken at 100 × magnification.

### Plasmid construction

pM20 contains the full-length B19V replicative from (RF) genome (nt 1–5596), and pN8 contains a half-ITR deleted B19 RF genome (nt 199–5410) [38,70]. They are diagramed in **Fig 2A**. pN8^mOriL^ and pN8^mOriR^ were constructed by mutating the STAT5BE of the *Ori* in the left and right half ITRs of the pN8, respectively. Both STAT5BE were mutated in pM20 and pN8 resulted in pM20^mOri^ and pN8^mOri^, respectively, which are diagramed with *mOri* shown, and the sequence of mutated *Ori* in the half right ITR is depicted (**Fig 6A**).

### Transfection

UT7/Epo-S1 cells were electroporated in V solution using Amaxa Nucleofector (Lonza, Basel, Switzerland), as described previously [25]. Briefly, B19V infectious clone pM20 or mutants were enzymatically digested with *Sal* I. The linearized DNA was gel-purified. 2 μg of DNA was used for electroporation of 2 × 10^6^ cells. After transfection, UT7/Epo-S1 cells were cultured under hypoxia of 1% O_2_.

### Flow cytometry and cell-cycle analysis

B19V-infected CD36^+^ EPCs were examined for virus infection by flow cytometry analysis with an anti-B19V capsid antibody, as described previously [25,50]. For cell-cycle analysis, a bromodeoxyuridine (BrdU) incorporation assay was used, as described previously [50].

### Southern blot analysis

Lower molecular DNA (Hirt DNA) was extracted from either B19V-infected CD36^+^ EPCs or transfected UT7/Epo-S1 cells by a Hirt extraction method, as described previously [45]. Hirt DNA extracted from UT7/Epo-S1 cells was further digested with *Dpn* I to remove non-replicated plasmid DNA input. Southern blot analysis was performed as reported previously [28,45]. B19V RF DNA M20 excised from pM20 with *Sal* I was used as a probe.

### Phosphorylated STAT5 protein purification

A biotinylated dsDNA probe [71], 5’-Bio-GAT ACT AGT TTC GTG GAA TCG TGG CAC TAT GAA CCA-3’, containing a STAT5BE (underlined), was synthesized by IDT (Coralville, IA) and used to purify pSTAT5, following a published protocol [72] with some modifications. Briefly, UT7/Epo-S1 cells grown in 14 dishes of 145 mm diameter were collected, washed with PBS, and resuspended in Lysis Buffer-1 (10 mM HEPES, pH 7.6, 0.1 mM EDTA, 1 mM DTT, 0.5% NP-40, 10 mM KCl, 0.5 mM PMSF, and protease inhibitor cocktail (PIC, MillopreSigma) for 5 min on ice. After vortexing, the lysate was centrifuged at 500 × g for 5 min at 4°C, and the nuclear pellet was washed with Lysis Buffer-1 without NP-40. The pellet was resuspended again in Lysis Buffer-2 (50 mM Tris, pH 7.6, 150 mM NaCl, 1 mM EDTA, 1% Triton X-100, 1 mM DTT, and 1 mM PMSF, and PIC), vortexed, and kept on ice for 30 min. The nuclear lysate was sonicated, centrifuged at 12,000 × g for 20 min, and then passed through a 0.45 μm filter before being mixed with streptavidin beads pre-bound with the biotin-dsDNA probe and incubated for several hours. The beads were then washed in Wash Buffer (50 mM Tris-HCl, pH 7.6, 150 mM NaCl, 3–4 μg/ml poly dI-dC (MilliporeSigma), 1 mM PMSF, and PIC). Bound proteins were eluted in Wash Buffer with increasing salt concentrations (0.3–1 M NaCl). The fractions containing pSTAT5 were identified by Western blotting.

### Electrophoretic mobility shift assay (EMSA)

Electrophoretic mobility shift assay (EMSA) was performed as previously reported [73]. Complementary forward and reverse oligonucleotides (synthesized at IDT, Coralville, IA) were annealed to form dsDNA probes, wt-Ori-39 and mut-Ori-39 that had the STAT5BE mutated [71] (**Fig 2B**). The probes were 5’ end labeled with ^32^P using [γ-^32^P] ATP. Each 20 μl binding reaction contained 3 μg/ml of poly dI-dC (MilliporeSigma).

### Chromatin immunoprecipitation (ChIP) assay

Chromatin immunoprecipitation (ChIP) assay was performed essentially as described previously [74,75] with modifications. Cells were fixed in 1% formaldehyde for 10 min at room temperature and then quenched in 125 mM glycine. Fixed cells were washed with PBS, and then lysed in 400 μl of Lysis Buffer (10 mM Tris-HCl, pH 8.0, 10 mM NaCl, 0.2% NP-40, 1 mM PMSF, and PIC) and incubated for 10 min on ice. After centrifugation at 2,500 rpm for 5 min at 4°C, the nuclear pellet was resuspended in 100 μl of Nuclear Lysis Buffer (50 mM Tris-HCl, pH 8.1,10 mM EDTA, 1% SDS, and PIC) for 10 min on ice. One ml IP Dilution Buffer (20 mM Tris-HCl, pH 8.1, 2 mM EDTA, 150 mM NaCl, 1% Triton X-100, and 0.01% SDS) was added, and chromatin was sheared by sonication at 80% power for 10 cycles of 15 s pulse and 1 min rest. Sonicated samples were centrifuged to remove debris, and the supernatant was split aliquots. Antibody (2.5 μg) was added to each aliquot, and the mixtures were incubated overnight at 4°C. For each sample, 10 μg of yeast tRNA was added to 40 μl of cold PBS-prewashed Protein A/G beads (Gold BioTechnology, Inc., St Louis, MO), and this mixture was added to the sample containing antibody and incubated with rocking for 6 h. Beads were collected by centrifugation and washed with IP Wash-1 (20 mM Tris, pH 8.1, 2 mM EDTA, 50 mM/500mM NaCl, 1% Triton X-100, 0.1% SDS) three times (first at low salt of 50 mM and then twice at 500 mM) for 10 min each at 4°C, followed by one wash with IP Wash-2 (10 mM Tris, pH 8.1, 1 mM EDTA, 0.25 M LiCl, 1% NP-40, and 1% deoxycholic acid) for 10 min at 4°C. The beads were then washed with cold TE, and protein-DNA complexes were eluted twice using 200 μl of Elution Buffer (100 mM sodium bicarbonate and 1% SDS) for 10 min at room temperature. Crosslinking was reversed by addition of 16 μl of 5 M NaCl and incubation at 65°C. DNA was purified with a Qiagen PCR purification kit (Qiagen, Hilden, Germany), and ChIP product was recovered in 50 μl of H_2_O, and used for PCR or quantitative PCR (qPCR) analysis.

### PCR or qPCR analysis

Immunoprecipitated viral DNA from ChIP assay was subjected to PCR analysis using either F1 and R1 or F1 and R2 primers spanning the viral origin region: F1 (nt 5036–5053), 5’-CCT GCC CCC TCC TAT ACC-3’, R1 (nt 5308–5285), 5’-CAG GAA ATG ACG TAA TTG TCC GCC-3’, and R2 (nt 5393–5376), 5’-ACG TCA ACC CCA AGC GCT-3’. q-PCR analysis was done as described previously [24], using the following primers: F (nt 353–378), 5’-GCA TCT GAT TTG GTG TCT TCT TTT AA-3’, R (421–403), 5’-TGG CTG CCC ATT TGC ATA A-3, and probe (nt 386–401), 5’ FAM-CGG GCT TTT TTC CCG C/IABkFQ-3’.

### Colony formation assay

The colony formation assay was performed with methyl cellulose-based medium (R&D Systems, Minneapolis, MN) according to the manufacturer’s instructions, with modifications. Briefly, CD36^+^ EPCs were cultured in Wong expansion medium and were treated with pimozide at various concentrations on Day 7. After 48 hours, ≥3 × 10^4^ cells from each well were cultured in semi-solid methyl cellulose-based medium for 10–12 days, at which time colony counts were assessed by someone who was blinded to the experimental conditions.

### Immunoprecipitation assay and Western blotting

Co-immunoprecipitation (Co-IP) assay was performed as previously described [73,76]. Briefly, UT7/Epo-S1 cells were collected, washed with PBS, and lysed in radioimmunoprecipitation assay (RIPA) buffer. After centrifugation at 12,000 rpm for 20 min at 4°C, supernatant was taken and split into aliquots. Each aliquot was incubated with 3 μg of an antibody of interest overnight at 4°C, and then 40 μl of Protein A/G beads (washed with ice-cold PBS three times beforehand) was added, followed by incubation for 6 h. The beads were collected by centrifugation and washed three to five times with 1 × PBS, and then resuspended in 1 × Laemmli sample buffer. Samples were boiled for 10 min and run on 10% SDS-polyacrylamide gels for Western blot analysis, which was performed as described previously [25,50,77]. Pull-down assay was performed similarly to Co-IP, except that anti-Flag-conjugated beads or control beads were used.

### Antibodies

The following primary antibodies were purchased: mouse anti-STAT5 (cat# sc-74442), rabbit anti-STAT5 (cat# sc-835), anti-STAT5A (cat# sc-271542) and anti-STAT5B (cat# sc-1656), anti-BrdU (IIB5) (cat# sc-32323) were from Santa Cruz (Dallas, TX); anti-MCM2 (cat# 12079), and anti-pSTAT5(Y694) (cat# 4322) were from Cell Signaling (Danvers, MA); anti-STAT5a/b pan-specific antibody (cat # AF2168) and normal IgG rabbit (cat# AB-105-C) were from R&D Systems Inc (Minneapolis, MN); anti-MCM5 antibody (cat# 2380–1) was from Epitomics (Burlingame, CA); anti-B19V capsid (cat# Mab8293) was from Millipore (Billerica, MA); anti-BrdU (clone B44) was from BD (Franklin Lakes, NJ); and anti-β-actin (cat# A5441) was from Sigma; anti-MCM3 (cat# A300-124A), anti-MCM5 (cat# A300-195A; for ChIP), and MCM7(cat#A300-128A) were from Bethyl Laboratories (Montgomery, TX); anti-ATM(pS1981) (cat#ab81292) were from Abcam (Cambridge, MA); and anti-ATR(pT1989) (cat#GTX128145) from GeneTex (Irvine, CA). Rat anti-NS1 polyclonal antibody was prepared in our lab as previously reported [25].

Horseradish peroxidase (HRP)-conjugated anti-mouse and anti-rabbit secondary antibodies were purchased from Sigma, and fluorescein isothiocyanate (FITC)-, Texas Red-, and Dylight405-conjugated anti-mouse, anti-rat, and anti-rabbit secondary antibodies were all purchased from Jackson ImmunoResearch (West Grove, PA).

### Statistics

Statistical analysis was performed using GraphPad Prism Version 7.0. Statistical significance was determined by using 1-way ANOVA analysis, followed by Tukey-Kramer post-test for comparison of three or more groups and unpaired (Student) t-test for comparison of two groups. Error bars show mean and standard deviation (Mean ± SD) unless otherwise specified.

## Supporting information

S1 Fig Pimozide abolishes B19V DNA replication in UT7/Epo-S1 cells. (A&B) Inhibition of B19V DNA replication. UT7/Epo-S1 cells were pre-incubated with DMSO or pimozide (at a final concentration of 10 μM or 20 μM), 6 h prior to M20 transfection. Transfected cells were cultured under hypoxic conditions. (A) At 48 h post-transfection, cells were collected for Hirt DNA extraction. The DNA samples were subjected to Southern blotting with a B19V M20 probe (upper panel). Mitochondrial (mito) DNA was probed as a loading control (lower panel). (B) At 48 h post-transfection, cells were collected for Western blotting with anti-pSTAT5(Y694). The blot was reprobed for β-actin. (C&D) Evaluation of the effect of pimozide on cell proliferation. CD36^+^ EPCs were treated with either DMSO or pimozide and then incubated with BrdU to perform a BrdU incorporation assay. (C) Results of a representative cell-cycle analysis. (D) Relative fold changes of the cell population in S-phase are shown with means and standard deviations, which were obtained from three independent experiments. P values are calculated using one-way ANOVA followed by Tukey-Kramer post-test, n.s. (P> 0.05) denotes no statistical significance.
(TIF)

S2 Fig Blockage of STAT5-DNA interaction inhibits B19V replication in UT7/Epo-S1 cells. (A) Southern blot analysis. UT7/Epo-S1 cells were incubated with either DMSO or STAT5-SH2 inhibitor (STAT5-SH2i; 250 μM or 500 μM) at 6 h prior to transfection, and then the cells were transfected with M20 DNA and cultured under hypoxic conditions. At 48 h post-transfection, cells were collected for Hirt DNA extraction. The DNA samples were subjected to Southern blotting with an M20 DNA probe. Mitochondrial (Mito) DNA was probed as a loading control (lower panel). (B&C) Evaluation of the effect of STAT5 inhibitor on cell proliferation. UT7/Epo-S1 cells were treated with either DMSO or STAT5-SH2i (at 250 μM or 500 μM), and then incubated with BrdU for BrdU incorporation assays. (B) Results of a representative cell-cycle analysis. (C) Relative fold changes of the cell population in S-phase are shown, with means and standard deviations shown. P values are calculated using one-way ANOVA followed by Tukey-Kramer post-test, compared with DMSO group. *** denotes P<0.001 and n.s. (P>0.05) for no statistical significance.
(TIF)

S3 Fig STAT5A is the major STAT5 isoform expressed in erythroid lineage cells. (A&B). Differential expression of STAT5A and STAT5B. (A) Cell lysates of UT7/Epo-S1 and EPCs were subjected to Western blotting with STAT5A/B pan-specific, STAT5A-specific, or STAT5B-specifc antibodies. Asterisks indicate dimerized or degraded or non-specific protein bands. (B) Purified STAT5 of UT7/Epo-S1 cells was subjected to Western blotting with a STAT5A/B pan-specific antibody. (C) Both STAT5A and STAT5B interact with the MCM2 complex. UT7/Epo-S1 cells were collected and lysed with RIPA buffer. The lysates were incubated with an anti-MCM2 or control IgG antibody for co-immunoprecipitation (Co-IP). Immunoprecipitated proteins were blotted for the presence of STAT5A, STAT5B, and MCM2 with anti-STAT5A, anti-STAT5B, and anti-MCM2 antibodies, respectively. The precipitated IgG heavy chain is also shown.
(TIF)

S4 Fig Analyses of MCM or STAT5 binding to B19V genome by ChIP assay. (A) UT7/Epo-S1 cells were transfected with M20 and allowed to replicate for 48 h under hypoxic conditions. Cells were collected for ChIP analysis. Anti-MCM2, anti-MCM3, anti-MCM5, anti-MCM7, and control IgG antibodies were used to pull down DNA-protein complex. Recovered DNA was analyzed by qPCR targeting the viral origin (Ori-qPCR). Error bars represent standard deviations taken from at least three experiments. P values were calculated using a Student’s t test, compared to the IgG control. ** P<0.01; * P<0.05. (B) A diagram of the Ori-qPCR amplicon targeting the viral replication origin (*Ori*) at the left ITR (L-ITR). The starting nucleotide numbers of both forward and reverse (F and R) and the location of the probe are indicated. (C) UT7/Epo-S1 cells were treated with either DMSO, STAT5-SH2i inhibitor (at 500 μM) or pimozide (at 15μM), as indicated in the figure, at 6h prior to transfection. Then, the cells were transfected with M20 and cultured under hypoxic conditions for 48 h. Cells were collected for ChIP analysis using an anti-STAT5 and Ori-qPCR. Error bars represent standard deviation taken from at least three experiments. P values were calculated using one-way ANOVA followed by Tukey-Kramer post-test, compared with the M20 group. **** denotes P<0.0001. (D) Mock- or M20- transfected UT7/Epo-S1 cells, cultured under hypoxic conditions for 48 h, were collected for ChIP assay using an anti-STAT5 antibody, followed by PCR using primers: forward (F, nt 3135–3156), 5’- GGA CTG TAG CAG ATG AAG AGC T-3’, and reverse (R, nt 3393–3373), 5’-GTG GCC CCC TCA CTC CAC AT-3’, primes as indicated in the diagram. Rabbit IgG was used as a negative control of pull-down, and M20 DNA was used as PCR positive control.
(TIF)

S5 Fig Pimozide does not affect hydroxyurea-induced ATR/ATM activation, and B19V infection did not alter pSTAT5 expression, in CD36^+^ EPCs. (A) CD36^+^ EPCs were treated with pimozide (at 15 μM). At 3 h post-treatment, cells were incubated with hydroxyurea at 10 mM for 24 h under hypoxic conditions. Then, the same numbers of the cells were collected for Western blot analysis of proteins, as indicated, using anti-ATM(pST1981), anti-ATR(pT1989), pSTAT5, and β-actin, respectively. (B) Mock and B19V-infected CD36^+^ EPCs, cultured under hypoxic conditions, were used for Western blot analysis of pSTAT5. The membrane was reprobed for β-actin.
(TIF)

S6 Fig pSTAT5 does not transactivate viral P6 promoter. (A) A diagram of lentivirus Lenti-ATF/P6-GFP. The virus was made as we reported previously [29]. (B) UT7/Epo-S1 (S1) cells or NS1-expressing UT7/Epo-S1 (NS1-S1) cells were transduced with Lenti-ATF/P6-GFP an MOI of 2–4 transduction units/cell and cultured under hypoxic conditions. At 24 h post-transduction, cells were incubated either with DMSO or pimozide (at a final concentration of 15 μM) for 48 h. Then, the cells were collected and subjected to flow cytometry analysis for a mean fluorescence intensity (MFI) value of GFP expression. P values are calculated using one-way ANOVA followed by Tukey-Kramer post-test, compared with DMSO group. **** denotes P<0.0001 and n.s. (P>0.05) for no statistical significance.
(TIF)

S7 Fig Pimozide and STAT5-SH2i synergistically inhibit B19V infection. CD36^+^ EPCs were pre-incubated with DMSO or pimozide (at 15 μM), STAT5-SH2i (at 250 μM), or pimozide plus STAT5-SH2i (at 15 and 250 μM, respectively), 6 h prior to B19V infection under hypoxic conditions. (A) At 48 h post-infection, cells were subjected to flow cytometry analysis using anti-B19V capsid antibody. Error bars represent standard deviation taken from at least three experiments. P values are calculated using one- way ANOVA followed by Tukey-Kramer post-test, compared with DMSO group. **** denotes P<0.0001. (B) Similarly, at 48 h post-treatment, uninfected cells were labeled with BrdU for cell cycle analysis. Numbers shown are percentages of cells at G1, S, and G2 phase, respectively.
(TIF)
