# Supplementary figures and images for: Phosphorylated STAT5 directly facilitates parvovirus B19 DNA replication in human erythroid progenitors through interaction with the MCM complex
Source: PLoS Pathog. 2017 May 1;13(5):e1006370. doi: 10.1371/journal.ppat.1006370 (PMC5426800; doi:10.1371/journal.ppat.1006370)

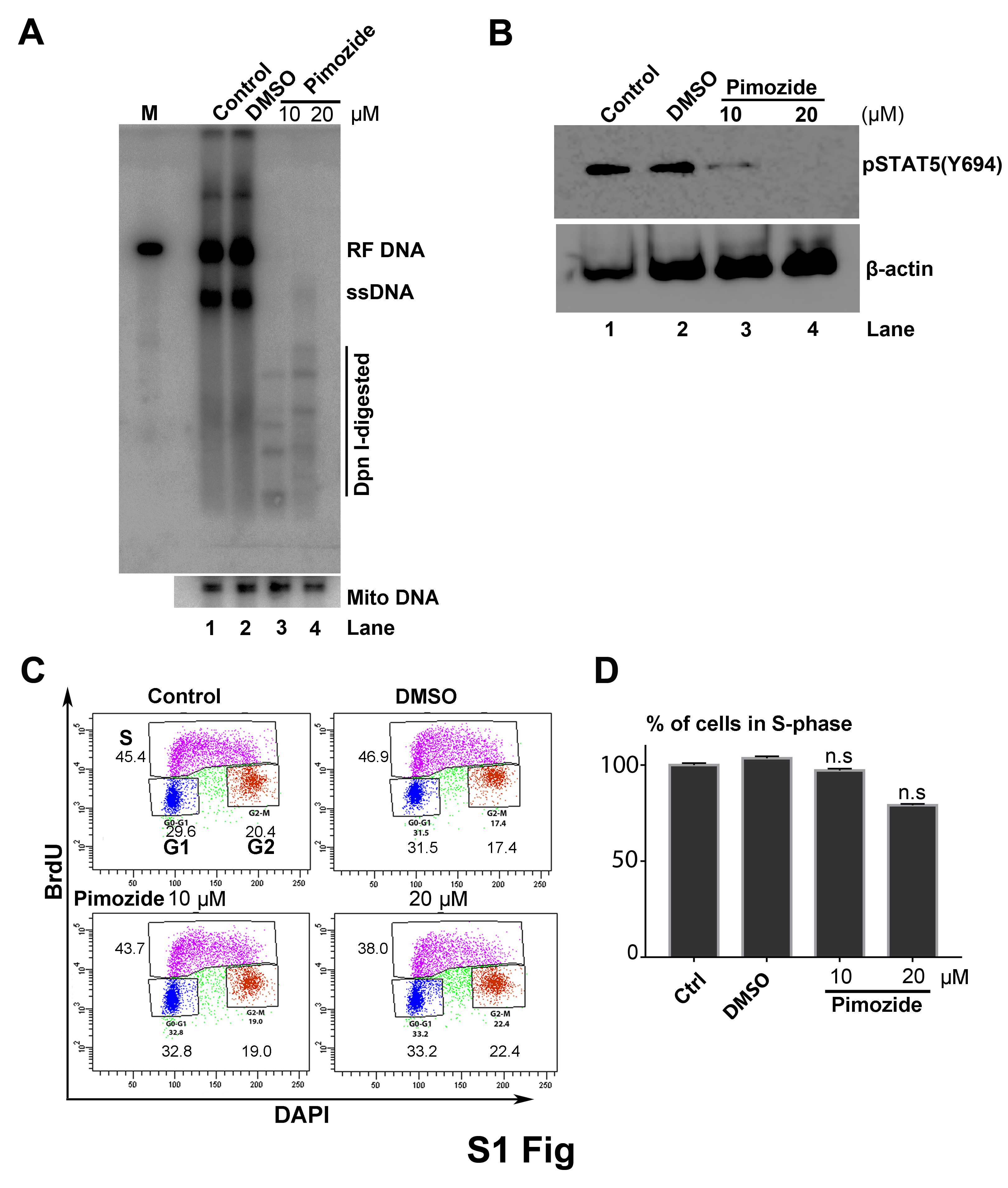

Supplement: S1 Fig — (A&B) Inhibition of B19V DNA replication. UT7/Epo-S1 cells were pre-incubated with DMSO or pimozide (at a final concentration of 10 μM or 20 μM), 6 h prior to M20 transfection. Transfected cells were cultured under hypoxic conditions. (A) At 48 h post-transfection, cells were collected for Hirt DNA extraction. The DNA samples were subjected to Southern blotting with a B19V M20 probe (upper panel). Mitochondrial (mito) DNA was probed as a loading control (lower panel). (B) At 48 h post-transfection, cells were collected for Western blotting with anti-pSTAT5(Y694). The blot was reprobed for β-actin. (C&D) Evaluation of the effect of pimozide on cell proliferation. CD36+ EPCs were treated with either DMSO or pimozide and then incubated with BrdU to perform a BrdU incorporation assay. (C) Results of a representative cell-cycle analysis. (D) Relative fold changes of the cell population in S-phase are shown with means and standard deviations, which were obtained from three independent experiments. P values are calculated using one-way ANOVA followed by Tukey-Kramer post-test, n.s. (P> 0.05) denotes no statistical significance. (TIF) [file ppat.1006370.s001.tif]

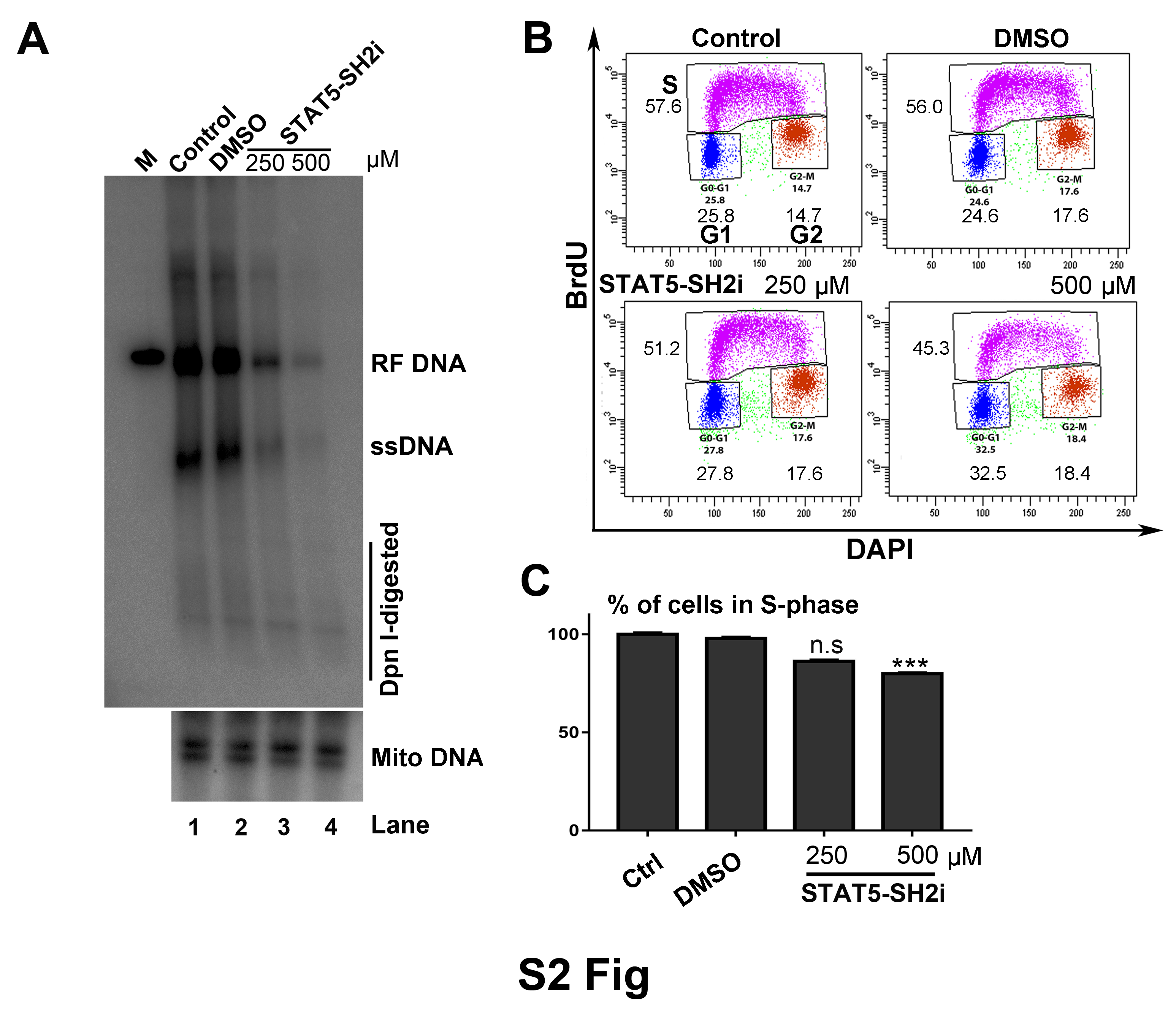

Supplement: S2 Fig — (A) Southern blot analysis. UT7/Epo-S1 cells were incubated with either DMSO or STAT5-SH2 inhibitor (STAT5-SH2i; 250 μM or 500 μM) at 6 h prior to transfection, and then the cells were transfected with M20 DNA and cultured under hypoxic conditions. At 48 h post-transfection, cells were collected for Hirt DNA extraction. The DNA samples were subjected to Southern blotting with an M20 DNA probe. Mitochondrial (Mito) DNA was probed as a loading control (lower panel). (B&C) Evaluation of the effect of STAT5 inhibitor on cell proliferation. UT7/Epo-S1 cells were treated with either DMSO or STAT5-SH2i (at 250 μM or 500 μM), and then incubated with BrdU for BrdU incorporation assays. (B) Results of a representative cell-cycle analysis. (C) Relative fold changes of the cell population in S-phase are shown, with means and standard deviations shown. P values are calculated using one-way ANOVA followed by Tukey-Kramer post-test, compared with DMSO group. *** denotes P<0.001 and n.s. (P>0.05) for no statistical significance. (TIF) [file ppat.1006370.s002.tif]

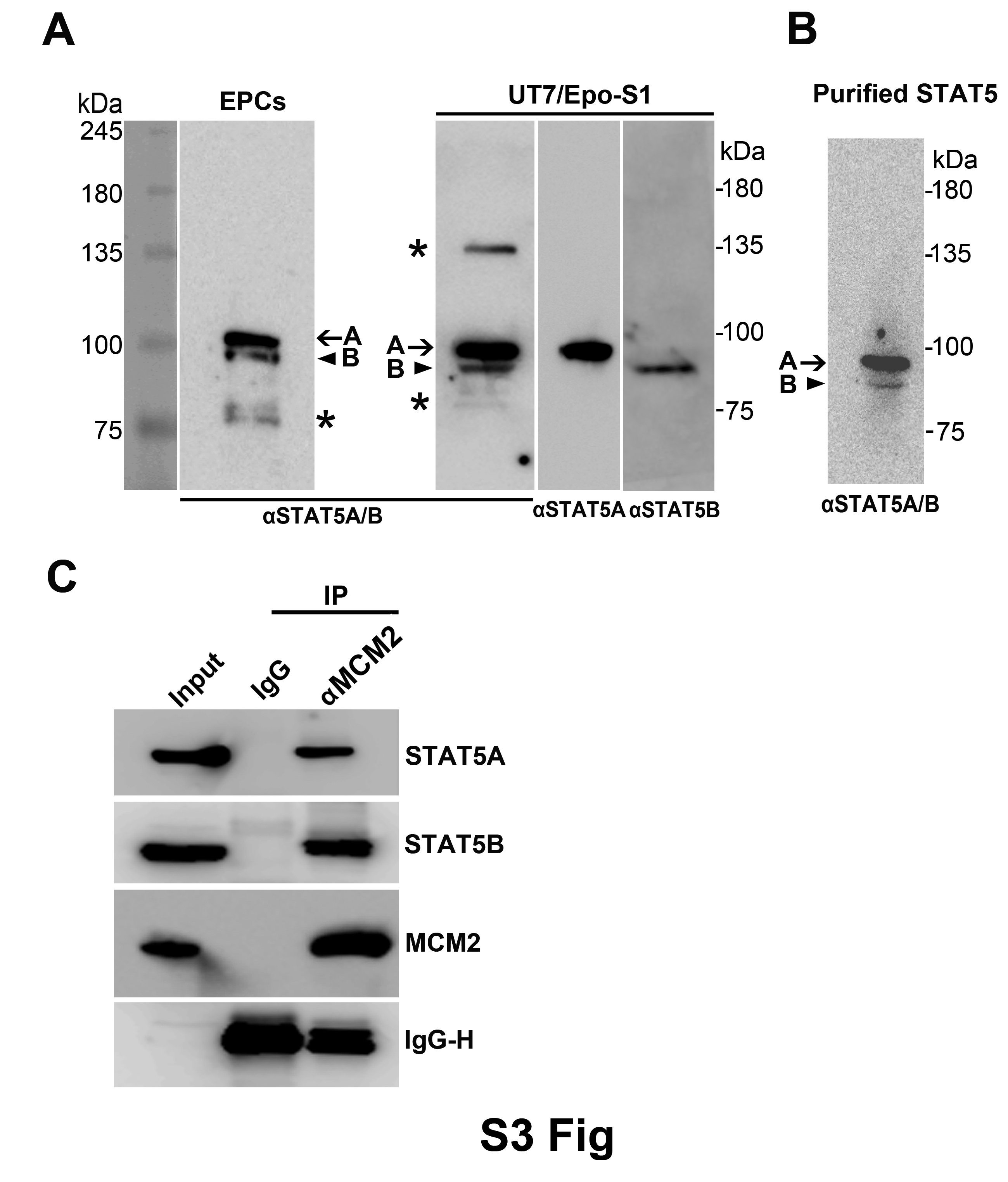

Supplement: S3 Fig — (A&B). Differential expression of STAT5A and STAT5B. (A) Cell lysates of UT7/Epo-S1 and EPCs were subjected to Western blotting with STAT5A/B pan-specific, STAT5A-specific, or STAT5B-specifc antibodies. Asterisks indicate dimerized or degraded or non-specific protein bands. (B) Purified STAT5 of UT7/Epo-S1 cells was subjected to Western blotting with a STAT5A/B pan-specific antibody. (C) Both STAT5A and STAT5B interact with the MCM2 complex. UT7/Epo-S1 cells were collected and lysed with RIPA buffer. The lysates were incubated with an anti-MCM2 or control IgG antibody for co-immunoprecipitation (Co-IP). Immunoprecipitated proteins were blotted for the presence of STAT5A, STAT5B, and MCM2 with anti-STAT5A, anti-STAT5B, and anti-MCM2 antibodies, respectively. The precipitated IgG heavy chain is also shown. (TIF) [file ppat.1006370.s003.tif]

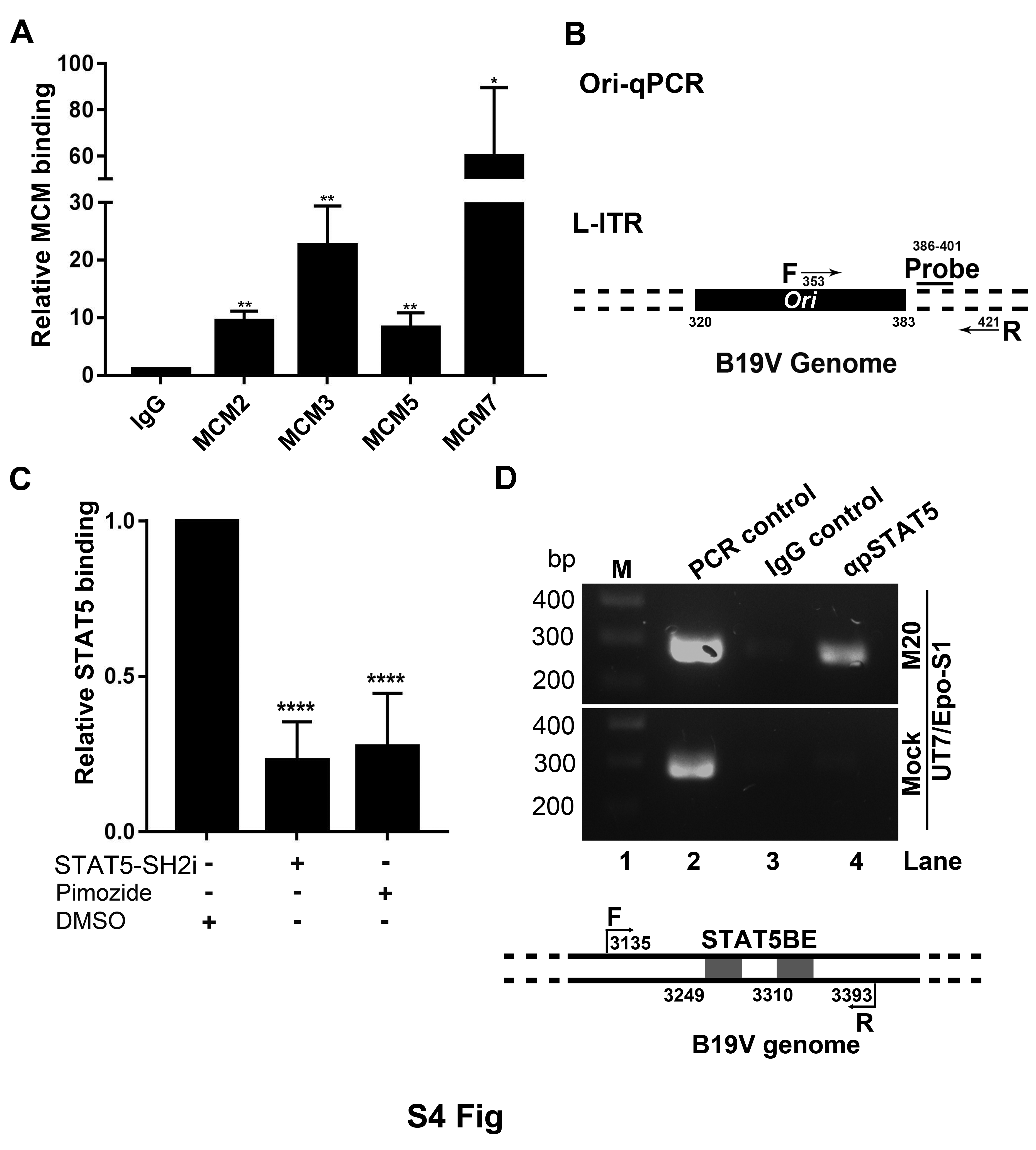

Supplement: S4 Fig — (A) UT7/Epo-S1 cells were transfected with M20 and allowed to replicate for 48 h under hypoxic conditions. Cells were collected for ChIP analysis. Anti-MCM2, anti-MCM3, anti-MCM5, anti-MCM7, and control IgG antibodies were used to pull down DNA-protein complex. Recovered DNA was analyzed by qPCR targeting the viral origin (Ori-qPCR). Error bars represent standard deviations taken from at least three experiments. P values were calculated using a Student’s t test, compared to the IgG control. ** P<0.01; * P<0.05. (B) A diagram of the Ori-qPCR amplicon targeting the viral replication origin (Ori) at the left ITR (L-ITR). The starting nucleotide numbers of both forward and reverse (F and R) and the location of the probe are indicated. (C) UT7/Epo-S1 cells were treated with either DMSO, STAT5-SH2i inhibitor (at 500 μM) or pimozide (at 15μM), as indicated in the figure, at 6h prior to transfection. Then, the cells were transfected with M20 and cultured under hypoxic conditions for 48 h. Cells were collected for ChIP analysis using an anti-STAT5 and Ori-qPCR. Error bars represent standard deviation taken from at least three experiments. P values were calculated using one-way ANOVA followed by Tukey-Kramer post-test, compared with the M20 group. **** denotes P<0.0001. (D) Mock- or M20- transfected UT7/Epo-S1 cells, cultured under hypoxic conditions for 48 h, were collected for ChIP assay using an anti-STAT5 antibody, followed by PCR using primers: forward (F, nt 3135–3156), 5’- GGA CTG TAG CAG ATG AAG AGC T-3’, and reverse (R, nt 3393–3373), 5’-GTG GCC CCC TCA CTC CAC AT-3’, primes as indicated in the diagram. Rabbit IgG was used as a negative control of pull-down, and M20 DNA was used as PCR positive control. (TIF) [file ppat.1006370.s004.tif]

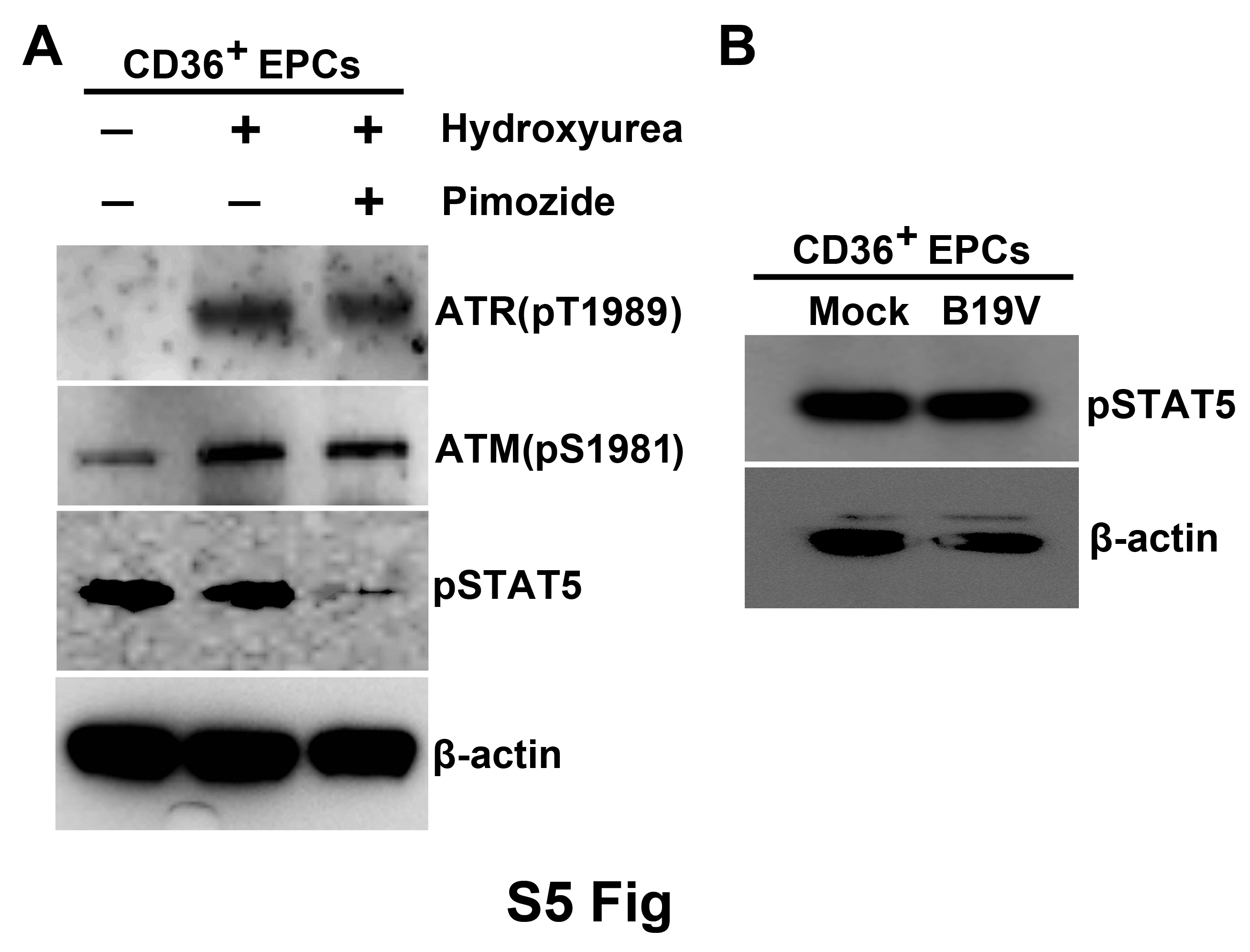

Supplement: S5 Fig — (A) CD36+ EPCs were treated with pimozide (at 15 μM). At 3 h post-treatment, cells were incubated with hydroxyurea at 10 mM for 24 h under hypoxic conditions. Then, the same numbers of the cells were collected for Western blot analysis of proteins, as indicated, using anti-ATM(pST1981), anti-ATR(pT1989), pSTAT5, and β-actin, respectively. (B) Mock and B19V-infected CD36+ EPCs, cultured under hypoxic conditions, were used for Western blot analysis of pSTAT5. The membrane was reprobed for β-actin. (TIF) [file ppat.1006370.s005.tif]

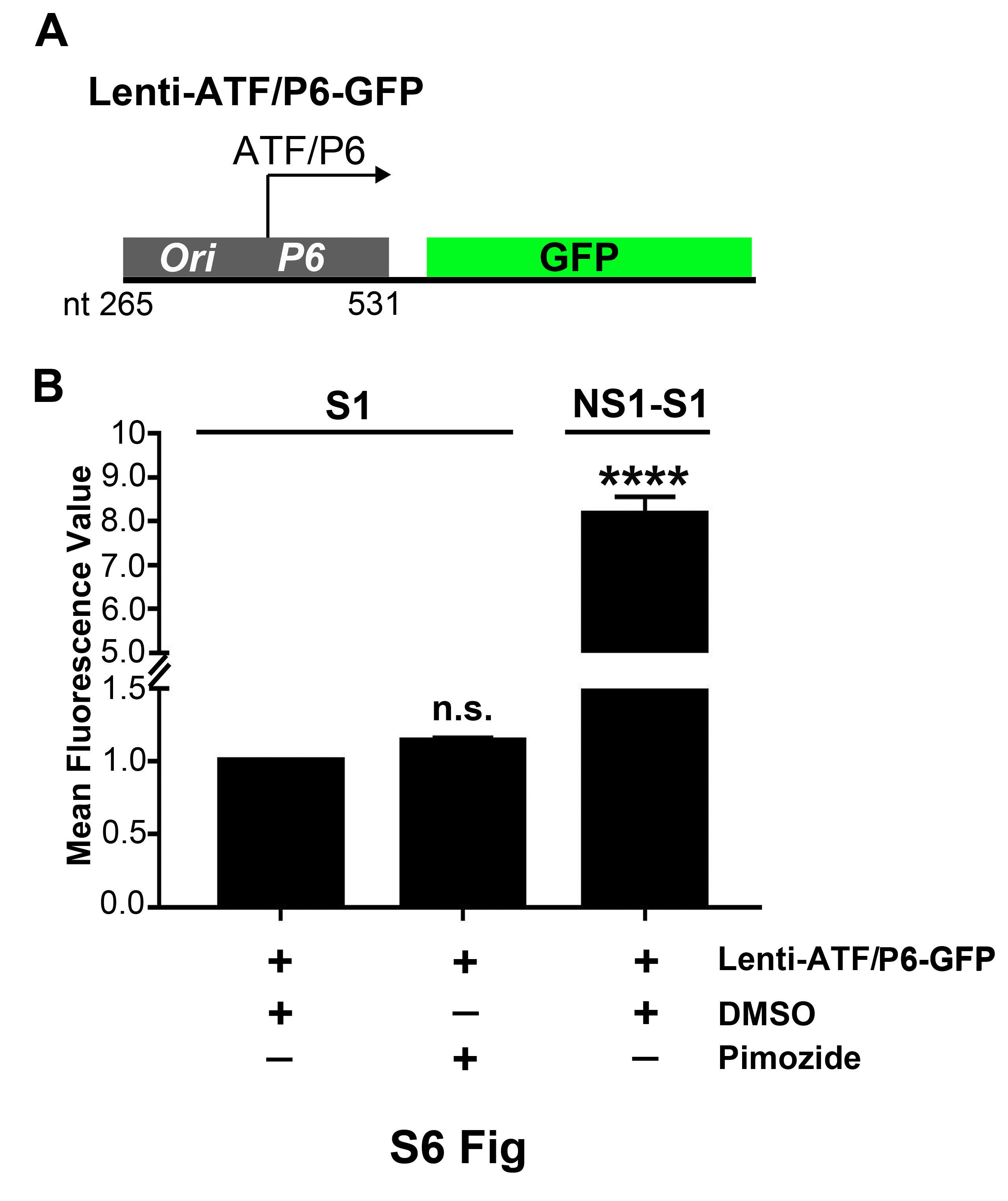

Supplement: S6 Fig — (A) A diagram of lentivirus Lenti-ATF/P6-GFP. The virus was made as we reported previously [29]. (B) UT7/Epo-S1 (S1) cells or NS1-expressing UT7/Epo-S1 (NS1-S1) cells were transduced with Lenti-ATF/P6-GFP an MOI of 2–4 transduction units/cell and cultured under hypoxic conditions. At 24 h post-transduction, cells were incubated either with DMSO or pimozide (at a final concentration of 15 μM) for 48 h. Then, the cells were collected and subjected to flow cytometry analysis for a mean fluorescence intensity (MFI) value of GFP expression. P values are calculated using one-way ANOVA followed by Tukey-Kramer post-test, compared with DMSO group. **** denotes P<0.0001 and n.s. (P>0.05) for no statistical significance. (TIF) [file ppat.1006370.s006.tif]

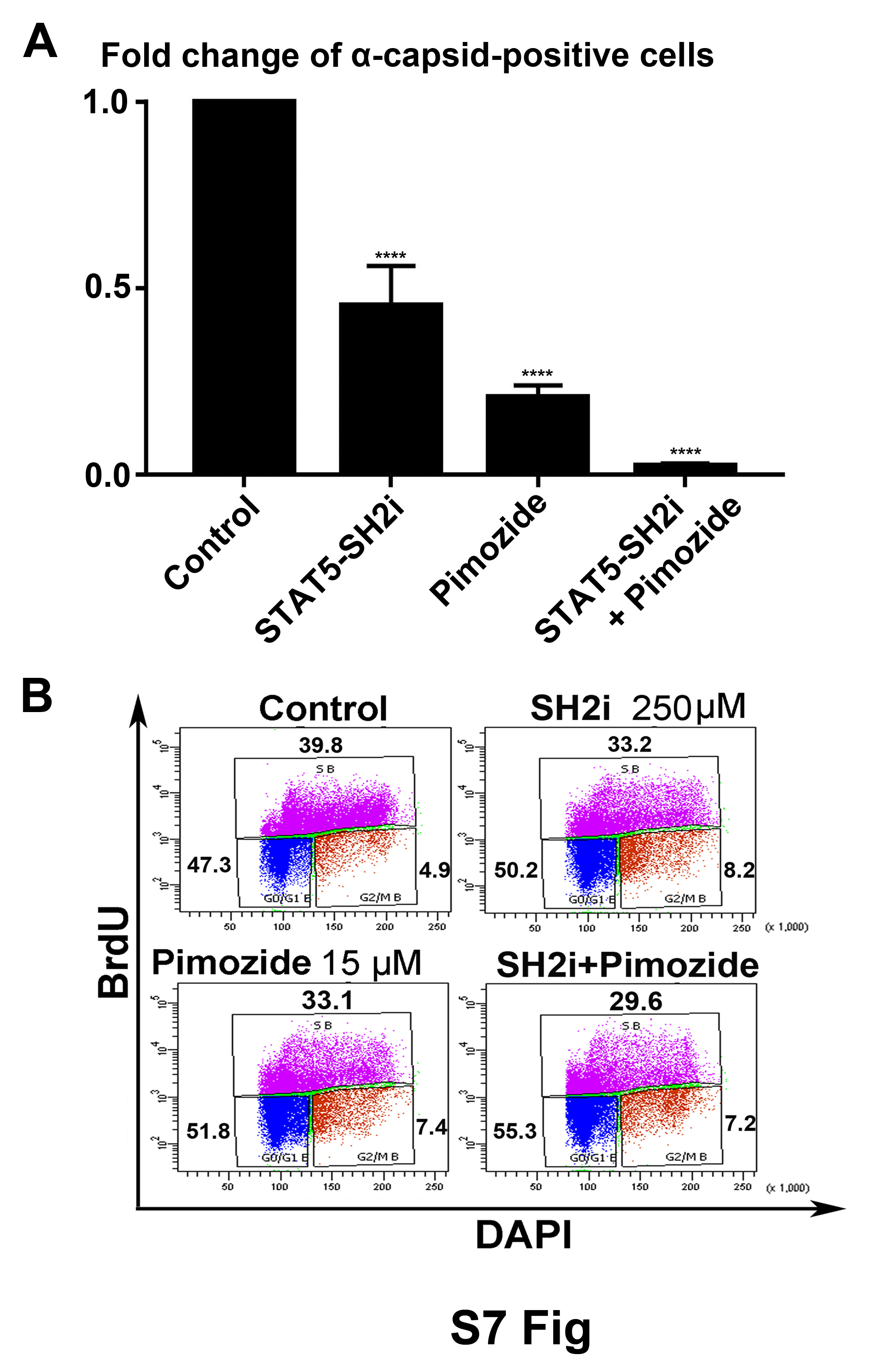

Supplement: S7 Fig — CD36+ EPCs were pre-incubated with DMSO or pimozide (at 15 μM), STAT5-SH2i (at 250 μM), or pimozide plus STAT5-SH2i (at 15 and 250 μM, respectively), 6 h prior to B19V infection under hypoxic conditions. (A) At 48 h post-infection, cells were subjected to flow cytometry analysis using anti-B19V capsid antibody. Error bars represent standard deviation taken from at least three experiments. P values are calculated using one- way ANOVA followed by Tukey-Kramer post-test, compared with DMSO group. **** denotes P<0.0001. (B) Similarly, at 48 h post-treatment, uninfected cells were labeled with BrdU for cell cycle analysis. Numbers shown are percentages of cells at G1, S, and G2 phase, respectively. (TIF) [file ppat.1006370.s007.tif]
